# The prognostic association of triglyceride-glucose index and its derived indicators with stable coronary artery disease patients undergoing percutaneous coronary intervention

**DOI:** 10.3389/fendo.2025.1465376

**Published:** 2025-01-22

**Authors:** Yu Shan, Maoning Lin, Fangfang Gu, Duanbin Li, Qiongjun Zhu, Zhezhe Chen, Wenbin Zhang, Guosheng Fu, Min Wang

**Affiliations:** ^1^ Department of Cardiology, Sir Run Run Shaw Hospital, College of Medicine, Zhejiang University, Hangzhou, Zhejiang, China; ^2^ Key Laboratory of Cardiovascular Intervention and Regenerative Medicine of Zhejiang Province, Hangzhou, China; ^3^ Department of Cardiology, The Affiliated Huzhou Hospital (Huzhou Central Hospital), College of Medicine, Zhejiang University, Huzhou, Zhejiang, China

**Keywords:** insulin resistance, triglyceride-glucose index, percutaneous coronary intervention, stable coronary artery disease, major adverse cardiovascular and cerebrovascular events

## Abstract

**Aims:**

Research on the triglyceride-glucose (TyG) index in patients with stable coronary artery disease (SCAD) remains relatively limited. Therefore, this study aims to investigate the association of the TyG index and its derived indicators, including the baseline TyG index, the baseline triglyceride glucose-body mass index (TyG-BMI), the mean TyG index, and the triglyceride glucose index-standard deviation (TyG-SD), with the prognosis of SCAD patients undergoing percutaneous coronary intervention (PCI).

**Methods:**

This retrospective study enrolled 2,306 patients. The Cox proportional hazards model was utilized to evaluate the association between the four TyG indicators and major adverse cardiovascular and cerebrovascular events (MACCE). The predictive ability of the four TyG indicators for MACCE was assessed using the time-dependent receiver operating characteristic (ROC) curve. Kaplan-Meier survival analysis was employed to assess the prognostic differences among groups.

**Results:**

After a median follow-up of 26.1 months, a total of 352 patients (15.3%) experienced MACCE. The Cox regression analysis revealed that under a fully adjusted model, when the four TyG indicators were stratified by tertiles, patients in the highest tertile of each TyG indicator had a significantly increased risk of MACCE compared to those in the lowest tertile. Specifically, the hazard ratio for baseline TyG index was 1.653 (95% confidence intervals (CI): 1.234-2.214), for baseline TyG-BMI was 2.467 (95%CI: 1.849-3.293), for mean TyG index was 2.451 (95%CI: 1.794-3.349), and for TyG-SD was 1.896 (95%CI: 1.430-2.513). Time-dependent ROC curve demonstrated that the mean TyG index had the strongest predictive ability for MACCE at each follow-up time point. The Kaplan-Meier analysis results showed that when the four TyG indicators were grouped by tertiles, there were significant differences in the cumulative incidence of MACCE among the three groups for each indicator (P < 0.05).

**Conclusion:**

Higher levels of the TyG index and its derived indicators were each independently and positively associated with the risk of MACCE in SCAD patients undergoing PCI. Among these indicators, the mean TyG index demonstrated the greatest predictive value for the risk of MACCE at each follow-up time point. Consequently, tracking the long-term trends of the TyG index may be prioritized in clinical practice.

## Graphical Abstract

SCAD, stable coronary artery disease; PCI, percutaneous coronary intervention; MACCE, major adverse cardiovascular and cerebrovascular events; TyG, triglyceride-glucose; TyG-BMI, triglyceride glucose-body mass index; TyG-SD, triglyceride glucose index-standard deviation.

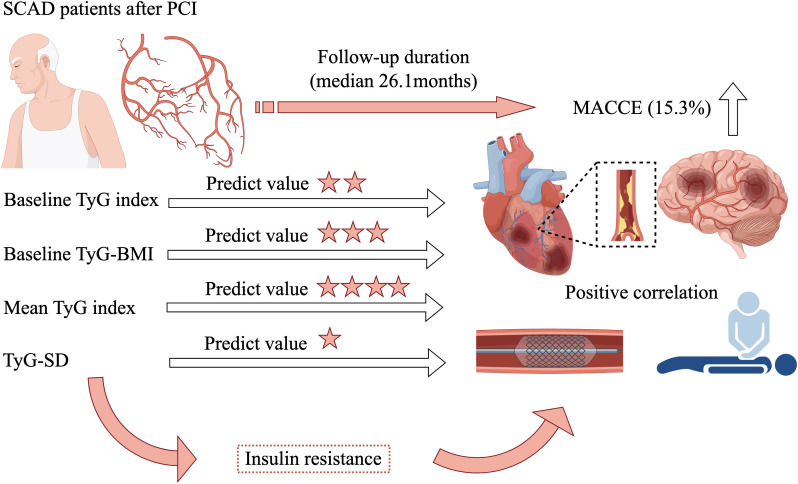
SCAD, stable coronary artery disease; PCI, percutaneous coronary intervention; MACCE, major adverse cardiovascular and cerebrovascular events; TyG, triglyceride-glucose; TyG-BMI, triglyceride glucose-body mass index; TyG-SD, triglyceride glucose index-standard deviation.

## Introduction

As a significant threat to global health, cardiovascular disease (CVD) tops the list of causes for death and healthcare expenditures, placing a considerable strain on healthcare systems across the globe (1, 2). Consequently, the timely identification of individuals at risk of poor CVD prognosis is of paramount importance.

Insulin resistance (IR) plays a crucial determinant in the development and prognosis of CVD (3, 4). Therefore, identifying reliable biomarkers for IR has long been a key focus of research (5). The triglyceride-glucose (TyG) index, derived from measurements of fasting blood glucose (FBG) and fasting triglycerides (TG), emerged as an innovative metric for detecting IR due to its convenience, low cost, and robust predictive capabilities (6–8). Moreover, TyG index also demonstrated commendable value in the occurrence and prognosis of CVD (9–11). However, with further investigation into the TyG index, researchers have discovered that derived indicators of the TyG index might more accurately reflect IR status than the baseline TyG index, and exhibited greater predictive value for CVD prognosis (12–14). For example, obesity is recognized as independently contributing to the progression of CVD (15). Numerous studies have proposed that combining the TyG index with body mass index (BMI) to form the triglyceride glucose-body mass index (TyG-BMI) showed a significantly stronger correlation with prognosis of CVD (16, 17). Furthermore, some researchers have pointed out that the TyG index also displayed dynamic changes over time (18, 19). A single baseline assessment of the TyG index may not fully reflect the long-term TyG index levels in individuals during extended follow-up. Consequently, long-term levels of the TyG index, derived from various time points (mean TyG index, and TyG index variability) could reasonably predict the prognosis of CVD (12, 20, 21). However, previous studies predominantly focused on exploring the correlation of baseline TyG index with the prognosis in acute coronary syndrome (ACS) patients (22–25). Research on the relationship between the TyG index and its derived indicators with the prognosis of stable coronary artery disease (SCAD) is still relatively scarce. Additionally, it remains unclear which of the various TyG index-derived indicators can provide better predictive value for the prognosis of SCAD. Therefore, the aim of this study was to investigate the relationship between the four TyG indicators (baseline TyG index, baseline TyG-BMI, mean TyG index, TyG index variability) and the prognosis of SCAD patients following percutaneous coronary intervention (PCI), while simultaneously evaluating which of these indicators could provide superior predictive value. The results of this study could contribute to the development of new strategies for enhancing the prognosis in SCAD patients, while offering valuable insights into the predictive significance of TyG index-derived indicators for clinical prognosis in this population.

## Methods

### Study population

**Figure 1:**
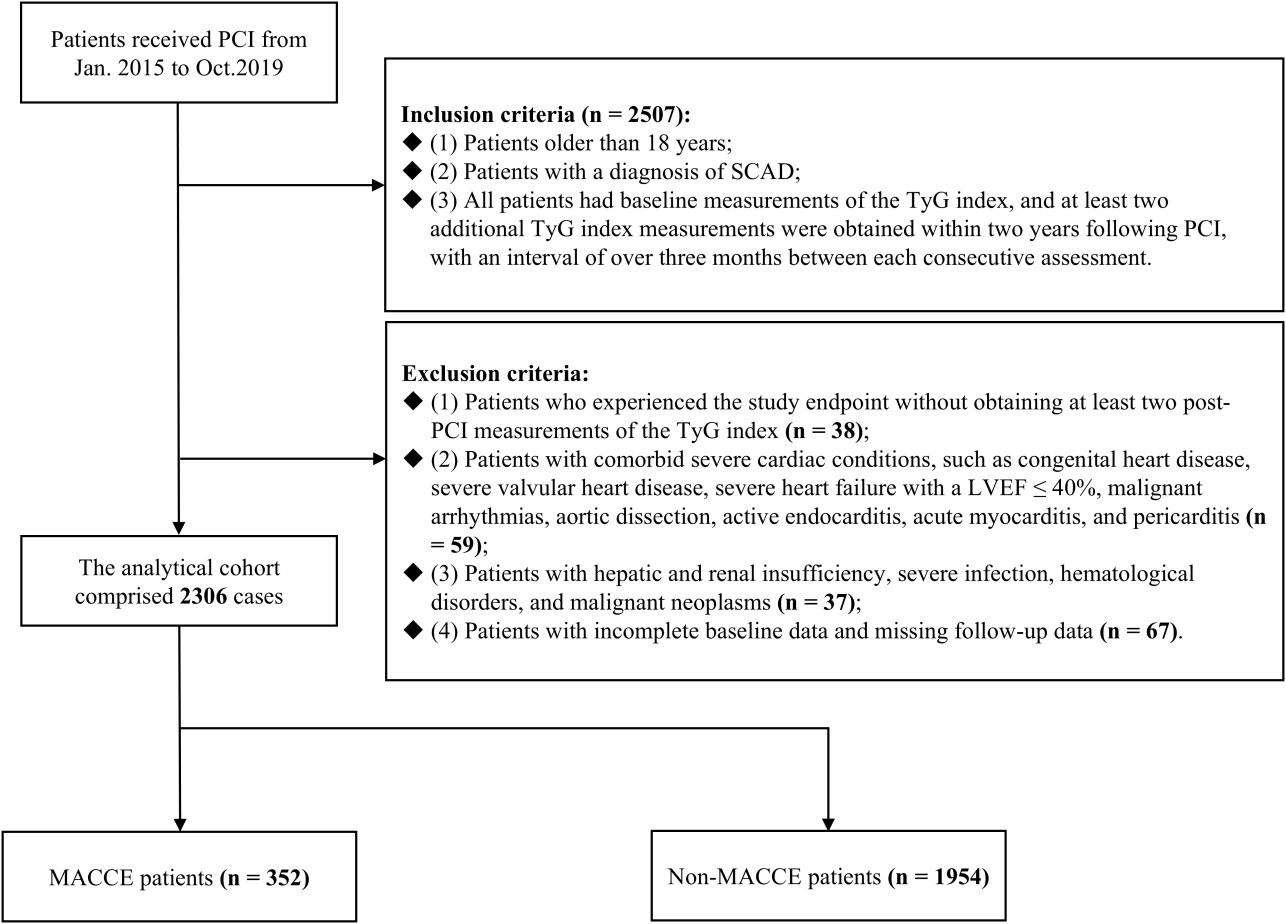
Flowchart of patient enrollment. PCI, percutaneous coronary intervention; SCAD, stable coronary artery disease; TyG, triglyceride-glucose; LVEF, left ventricular ejection fraction; MACCE, major adverse cardiovascular and cerebrovascular events.

From January 2015 to October 2019, patients with SCAD who underwent PCI at Sir Run Run Shaw Hospital, affiliated with Zhejiang University School of Medicine, were the subjects of a retrospective analysis conducted in the study. Adhering to the Declaration of Helsinki, the study received approval from the Sir Run Run Shaw Hospital Ethics Committee (approval number 2020-591-03). **Figure 1** illustrated the patient screening process, culminating in the inclusion of 2,306 patients who met the subsequent criteria for inclusion and exclusion.

The following outlines the criteria used for participant inclusion (n = 2507) (1): Patients older than 18 years (2). Patients with a diagnosis of SCAD (3). All patients had baseline measurements of the TyG index, and at least two additional TyG index measurements were obtained within two years following PCI, with an interval of over three months between each consecutive assessment. The criteria for participant exclusion were specified below: (1) Patients who experienced the study endpoint without obtaining at least two post-PCI measurements of the TyG index (n = 38). (2) Patients with comorbid severe cardiac conditions, such as congenital heart disease, severe valvular heart disease, severe heart failure with a left ventricular ejection fraction (LVEF) ≤ 40%, malignant arrhythmias, aortic dissection, active endocarditis, acute myocarditis, and pericarditis (n = 59). (3) Patients with hepatic and renal insufficiency, severe infection, hematological disorders, and malignant neoplasms (n = 37). (4) Patients with incomplete baseline data and missing follow-up data (n = 67).

### Definitions

In adherence to the 2013 European Society of Cardiology guidelines, SCAD encompasses patients presenting with recurrent, transient ischemic episodes stemming from an imbalance between oxygen supply and demand, within the context of established coronary artery stenosis—including both stable angina and silent ischemia—and those who have reached a state of stabilization subsequent to ACS (26). The calculation of the TyG index was based on the formula: TyG index = ln [TG (mg/dL) × FBG (mg/dL)/2] (12). The TyG-BMI calculation was based on the following formula: TyG-BMI = TyG index × BMI (kg/m^2^) (16). BMI was calculated as weight (kg)/height (m)^2^ (16). The mean TyG index was ascertained by computing the arithmetic average of all TyG index recordings for each subject. The variability of the TyG index was determined based on the standard deviation (SD) of all TyG index recordings for each subject, which was denoted as the triglyceride glucose index-standard deviation (TyG-SD). Multivessel lesion was characterized by the presence of at least two epicardial coronary arteries or their major branches with luminal narrowing of 50% or greater, and/or the presence of left main stem disease (27). Hypertension was defined as having a systolic blood pressure ≥ 140 mmHg or a diastolic blood pressure ≥ 90 mmHg, along with either a prior diagnosis of hypertension or the use of medications designed to manage blood pressure (21). Diabetes was identified through meeting any of the following criteria: FBG ≥ 7.0mmol/L, glycated hemoglobin A1c (HbA1c) ≥ 6.5%, treatment with oral hypoglycemic drugs or insulin therapy, or a documented history of diabetes (28). The smoking status was delineated into three categories: never, former, and current. Drinking status was also categorized into three groups: never, former, and current.

### Data collection

In this study, a meticulous review and organization of patient data was undertaken, encompassing the collection of demographic information, medical history, laboratory and echocardiographic indicators, pharmaceutical usage, and detailed information on PCI, all meticulously documented by physicians with extensive clinical experience and rigorous training. Furthermore, after a period of at least 8 hours of fasting, venous blood was drawn for the measurement of FBG, TG, low-density lipoprotein cholesterol (LDL-C), HbA1c, and other biochemical parameters.

### Follow-up endpoint

The study conducted follow-up assessments over a median duration of 26.1 months. Patients were followed up through a variety of methods, including in-person consultations, telephonic communications, and the administration of questionnaires. The endpoint of this study was major adverse cardiovascular and cerebrovascular events (MACCE), a composite metric that specifically encompasses non-fatal stroke, non-fatal acute myocardial infarction (AMI), target vessel revascularization (TVR), and all-cause mortality. Stroke was recognized by the presence of International Classification of Diseases (ICD)-10 codes I60 to I61, as well as code I63, while AMI was identified through the documentation of ICD-10 codes I21. TVR was characterized by any unforeseen revascularization procedures involving either the target or nontarget coronary arteries, encompassing both coronary artery bypass grafting and PCI (29). Death from any cause was referred to as all-cause mortality.

### Statistical analysis

Expressed in terms of the mean with SD as well as median and interquartile range, the continuous variables were outlined. Student’s t-test was the chosen method for paired comparisons when data exhibited a normal or near-normal distribution. Conversely, for data that followed a non-parametric distribution, the paired comparisons were conducted using the Mann-Whitney U-test. Case numbers and their corresponding percentages were used to illustrate the categorical variables, and the variations among them were assessed using Fisher’s exact test or χ² test.

This study stratified participants into tertiles based on four TyG indicators (baseline TyG index, baseline TyG-BMI, mean TyG index, TyG-SD), with the designation Q1 < Q2 < Q3, using Q1 as the reference group. The relationship between these four TyG indicators and the potential for MACCE in SCAD patients undergoing PCI was evaluated, employing the Cox proportional hazards model. In addition, the four TyG indicators were also analyzed as continuous variables in Cox regression models. Hazard ratios (HR), accompanied by their respective 95% confidence intervals (CI), were utilized to convey the results. Three hazard models were constructed based on established influencing factors from previous studies (30–33). Model 1 incorporated adjustments for age and gender (male or female). Model 2 incorporated adjustments for age, gender, baseline BMI, smoke (never, former, and current), drink (never, former, and current), hypertension (yes or no), and diabetes (yes or no). Model 3, the fully adjusted model, was adjusted for age, gender, baseline BMI, smoke, drink, hypertension, and diabetes, prior AMI (yes or no), baseline LDL-C, multivessel lesion (yes or no), LVEF, and medications (administration of angiotensin converting enzyme inhibitor/angiotensin receptor blocker, antidiabetic agents, antihypertensive agents, and β-receptor blocker) (yes or no). Given the significant collinearity observed between the baseline TyG-BMI and the baseline BMI, the model employed at baseline TyG-BMI excludes the baseline BMI as a variable. The study utilized time-dependent receiver operating characteristic (ROC) curves to evaluate the predictive capacity of four TyG indicators at various follow-up time points (12, 18, 24, 30, 36, and 42 months) for MACCE and to calculate the Youden index and the cut-off point (34, 35). Additionally, the study further employed the Kaplan-Meier survival analysis and Log-rank test to examine the prognostic disparities among groups categorized by the tertiles of the four TyG indicators. Finally, the study stratified participants based on gender (male or female), diabetes (yes or no), hypertension (yes or no), and LDL-C levels (<1.8mmol/L or ≥1.8mmol/L) to investigate whether the correlation of four TyG indicators with MACCE varies across different populations.

Statistical significance was determined by a threshold of P < 0.05, employing a two-tailed test. The dataset was analyzed and evaluated through the application of the SPSS software suite, specifically edition 23.0, an IBM product hailing from Chicago, Illinois. Furthermore, the analytical process was complemented by the utilization of R, version 4.3.0, which emanates from Vienna, Austria.

## Results

### Baseline characteristics and patient screening

A cumulative number of 2,306 SCAD individuals who underwent PCI were ultimately enrolled in this study. **Table 1** presented an exhaustive overview of the key baseline demographic and clinical features. The study cohort had an average age of 64.44 ± 10.15 years, with 68.8% males. Within this population, 352 individuals developed MACCE subsequent to PCI. Of these, 197 patients underwent TVR, 53 individuals suffered a non-fatal AMI, 43 patients experienced a non-fatal stroke, and 59 patients experienced an all-cause mortality. Among MACCE patients, significantly higher proportions were observed for hypertension (72.7% vs. 64.8%, P = 0.004), diabetes (41.5% vs. 23.9%, P < 0.001), prior stroke (11.1% vs. 7.5%, P = 0.024), and multivessel disease (16.5% vs. 12.1%, P = 0.023). Moreover, these patients also exhibited higher levels of BMI, white blood cell, FBG, TG, baseline TyG index, baseline TyG-BMI, mean TyG index, TyG-SD, HbA1c, C-reactive protein, and LDL-C (P < 0.05).

**Table 1:** Baseline characteristics of the two groups.

	Overall (n=2306)	MACCE		*P* value
		No (n=1954)	Yes (n=352)	
Demographic features				
Age, yrs	64.44 ± 10.15	64.16 ± 10.06	65.98 ± 10.53	0.002*
Male, n (%)	1587 (68.8)	1341 (68.6)	246 (69.9)	0.639
Hypertension, n (%)	1522 (66.0)	1266 (64.8)	256 (72.7)	0.004*
Diabetes, n (%)	613 (26.6)	467 (23.9)	146 (41.5)	<0.001*
Smoke, n (%)				0.294
Never	637 (27.6)	551 (28.2)	86 (24.4)	
Former	764 (33.1)	638 (32.7)	126 (35.8)	
Current	905 (39.2)	765 (39.2)	140 (39.8)	
Drink, n (%)				0.144
Never	1080 (46.8)	928 (47.5)	152 (43.2)	
Former	588 (25.5)	484 (24.8)	104 (29.5)	
Current	638 (27.7)	542 (27.7)	96 (27.3)	
Prior stroke, n (%)	186 (8.1)	147 (7.5)	39 (11.1)	0.024*
Prior AMI, n (%)	130 (5.6)	111 (5.7)	19 (5.4)	0.832
Prior PCI, n (%)	165 (7.2)	134 (6.9)	31 (8.8)	0.192
Family history of CAD, n (%)	357 (15.5)	293 (15.0)	64 (18.2)	0.128
BMI, kg/m^2^	24.29 ± 3.32	24.13 ± 3.22	25.14 ± 3.72	<0.001*
LVEF, %	65.81 ± 8.17	65.98 ± 8.12	64.92 ± 8.36	0.025*
Laboratory information				
White blood cell, ×10^9^/L	6.46 ± 2.07	6.39 ± 2.03	6.80 ± 2.27	0.001*
FBG, mmol/L	6.40 ± 2.51	6.25 ± 2.29	7.20 ± 3.37	<0.001*
TG, mmol/L	1.38 [1.01. 1.95]	1.36 [0.99. 1.91]	1.52 [1.11. 2.17]	<0.001*
Baseline TyG index	8.85 ± 0.63	8.81 ± 0.59	9.07 ± 0.78	<0.001*
Baseline TyG-BMI	215.39 ± 36.29	213.00 ± 34.67	228.69 ± 41.82	<0.001*
Mean TyG index	8.76 ± 0.51	8.72 ± 0.48	9.01 ± 0.58	<0.001*
TyG-SD	0.304 [0.203, 0.438]	0.295 [0.198, 0.421]	0.373 [0.252, 0.531]	<0.001*
HbA1c, %	6.23 ± 1.10	6.16 ± 1.03	6.60 ± 1.36	<0.001*
TC, mmol/L	4.21 ± 1.25	4.18 ± 1.20	4.36 ± 1.48	0.017*
LDL-C, mmol/L	2.23 ± 0.94	2.20 ± 0.92	2.39 ± 1.04	<0.001*
NT-proBNP, pg/mL	139.5 [50.0, 376.0]	131.0 [48.0, 367.0]	233.5 [64.8, 455.8]	<0.001*
eGFR, ml/(min×1.73 m^2^)	86.75 ± 20.68	87.02 ± 20.36	85.21 ± 22.40	0.229
C-reactive protein, mg/L	1.50 [0.70, 3.60]	1.40 [0.60, 3.40]	2.10 [1.00, 5.93]	<0.001*
PCI procedure data				
Multivessel lesion, n (%)	294 (12.7)	236 (12.1)	58 (16.5)	0.023*
Total length of stents, mm	30.0 [20.0, 48.0]	30.0 [20.0, 48.0]	31.0 [23.0, 51.0]	0.003*
Medication				
Antidiabetic agents, n (%)	551 (23.9)	435 (22.3)	116 (33.0)	<0.001*
Antihypertensive agents, n (%)	1466 (63.6)	1215 (62.2)	251 (71.3)	0.001*
Aspirin, n (%)	2239 (97.1)	1898 (97.1)	341 (96.9)	0.790
Clopidogrel/Ticagrelor, n (%)	2246 (97.4)	1906 (97.5)	340 (96.6)	0.301
Statin, n (%)	2274 (98.6)	1929 (98.7)	345 (98.0)	0.295
β-receptor blocker, n (%)	933 (40.5)	778 (39.8)	155 (44.0)	0.138
ACEI/ARB, n (%)	1098 (47.6)	918 (47.0)	180 (51.1)	0.151

Categorical variables were presented as counts alongside their respective percentages, whereas continuous variables were depicted as the mean with the standard deviation or the median along with the interquartile range. MACCE, major adverse cardiovascular and cerebrovascular events; AMI, acute myocardial infarction; PCI, percutaneous coronary intervention; CAD, coronary artery disease; BMI, body mass index; LVEF, left ventricular ejection fraction; FBG, fasting blood glucose; TG, triglycerides; TyG, triglyceride-glucose; TyG-BMI, triglyceride glucose-body mass index; TyG-SD, triglyceride glucose index-standard deviation; HbA1c, glycosylated hemoglobin A1c; TC, total cholesterol; LDL-C, low density lipoprotein cholesterol; NT-proBNP, N-terminal pro-B-type natriuretic peptide; eGFR, estimated glomerular filtration rate; ACEI, angiotensin converting enzyme inhibitor; ARB, angiotensin receptor blocker; *P < 0.05.

### Association between the four TyG indicators and MACCE

Association between the four TyG indicators and the MACCE risk across varying models was illustrated in **Table 2**. When analyzed as continuous variables, all four TyG indicators demonstrated an independent positive correlation with the risk of MACCE in each model (P < 0.05). Moreover, in all three models, the highest tertile levels of the four TyG indicators were also consistently and significantly positively associated with MACCE risk (P < 0.05). In Model 3, when baseline TyG index was categorized into tertiles, MACCE risk was significantly higher in the Q2 and Q3 groups compared to the Q1 group (Q2 HR: 1.409, 95% CI: 1.049-1.893; Q3 HR: 1.653, 95% CI: 1.234-2.214). Similarly, when baseline TyG-BMI was categorized, MACCE risk for the Q2 and Q3 groups was also relatively higher compared to the Q1 group (Q2 HR: 1.561, 95% CI: 1.152-2.116; Q3 HR: 2.467, 95% CI: 1.849-3.293). Moreover, when mean TyG index was utilized as a categorical variable, MACCE risk in the Q3 group was 2.451-fold (95% CI: 1.794-3.349) higher than the Q1 group. When TyG-SD was categorized, MACCE risk in the Q3 group was 1.896-fold (95% CI: 1.430-2.513) higher than the Q1 group.

**Table 2:** Hazard ratios for MACCE based on baseline TyG Index, baseline TyG-BMI, Mean TyG index, and TyG-SD.

Variables	Events/Overall (%)	Model 1	*P* value	Model 2	*P* value	Model 3	*P* value
		Adjusted HR (95%CI)		Adjusted HR (95%CI)		Adjusted HR (95%CI)	
Baseline TyG index		1.800 (1.558-2.080)	<0.001*	1.521 (1.299-1.782)	<0.001*	1.499 (1.275-1.764)	<0.001*
Q1 ≤ 8.53	79/769 (10.3)	1 (Reference)		1 (Reference)		1 (Reference)	
Q2 < 8.53, ≤ 9.05	116/769 (15.1)	1.677 (1.257-2.236)	<0.001*	1.439 (1.074-1.929)	0.015*	1.409 (1.049-1.893)	0.023*
Q3 > 9.05	157/768 (20.4)	2.302 (1.752-3.025)	<0.001*	1.740 (1.307-2.318)	<0.001*	1.653 (1.234-2.214)	0.001*
*P* for trend			<0.001*		<0.001*		<0.001*
Baseline TyG-BMI		1.011 (1.009-1.014)	<0.001*	1.010 (1.007-1.012)	<0.001*	1.009 (1.007-1.012)	<0.001*
Q1 ≤ 198.12	70/769 (9.1)	1 (Reference)		1 (Reference)		1 (Reference)	
Q2 < 198.12, ≤ 228.69	108/769 (14.0)	1.701 (1.257-2.301)	0.001*	1.577 (1.163-2.137)	0.003*	1.561 (1.152-2.116)	0.004*
Q3 > 228.69	174/768 (22.7)	2.917 (2.204-3.861)	<0.001*	2.524 (1.895-3.361)	<0.001*	2.467 (1.849-3.293)	<0.001*
*P* for trend			<0.001*		<0.001*		<0.001*
Mean TyG index		2.567 (2.152-3.062)	<0.001*	2.114 (1.738-2.572)	<0.001*	2.128 (1.741-2.601)	<0.001*
Q1 ≤ 8.52	61/769 (7.9)	1 (Reference)		1 (Reference)		1 (Reference)	
Q2 < 8.52, ≤ 8.92	114/769 (14.8)	2.005 (1.468-2.738)	<0.001*	1.790 (1.307-2.453)	<0.001*	1.746 (1.272-2.397)	0.001*
Q3 > 8.92	177/768 (23.0)	3.300 (2.460-4.425)	<0.001*	2.504 (1.837-3.413)	<0.001*	2.451 (1.794-3.349)	<0.001*
*P* for trend			<0.001*		<0.001*		<0.001*
TyG-SD		2.972 (2.264-3.900)	<0.001*	2.429 (1.771-3.330)	<0.001*	2.547 (1.839-3.530)	<0.001*
Q1 ≤ 0.2375	75/769 (9.8)	1 (Reference)		1 (Reference)		1 (Reference)	
Q2 < 0.2375, ≤ 0.3847	111/769 (14.4)	1.523 (1.135-2.042)	0.005*	1.416 (1.055-1.900)	0.021*	1.411 (1.050-1.896)	0.022*
Q3 > 0.3847	166/768 (21.6)	2.276 (1.732-2.991)	<0.001*	1.902 (1.438-2.517)	<0.001*	1.896 (1.430-2.513)	<0.001*
*P* for trend			<0.001*		<0.001*		<0.001*

Model 1: Adjusted for age and gender (male or female).

Model 2: Adjusted for age, gender (male or female), baseline BMI, smoke (never, former, and current), drink (never, former, and current), hypertension (yes or no), and diabetes (yes or no).

Model 3: Adjusted for age, gender (male or female), baseline BMI, smoke (never, former, and current), drink (never, former, and current), hypertension (yes or no), and diabetes (yes or no), prior AMI (yes or no), baseline LDL-C, multivessel lesion (yes or no), LVEF, and medications (administration of ACEI/ARB, antidiabetic agents, antihypertensive agents, and β-receptor blocker) (yes or no).

Given the significant collinearity observed between the TyG-BMI and the BMI, the model employed at baseline TyG-BMI excludes the BMI as a variable. MACCE, major adverse cardiovascular and cerebrovascular events; TyG, triglyceride-glucose; TyG-BMI, triglyceride glucose-body mass index; TyG-SD, triglyceride glucose index-standard deviation; HR, hazard ratios; CI, confidence interval; BMI: body mass index; AMI, acute myocardial infarction; LDL-C, low density lipoprotein cholesterol; LVEF, left ventricular ejection fraction; ACEI, angiotensin converting enzyme inhibitor; ARB, angiotensin receptor blocker; *P < 0.05.

### The disparities in MACCE risk among tertiles of the four TyG indicators

**Figure 2:**
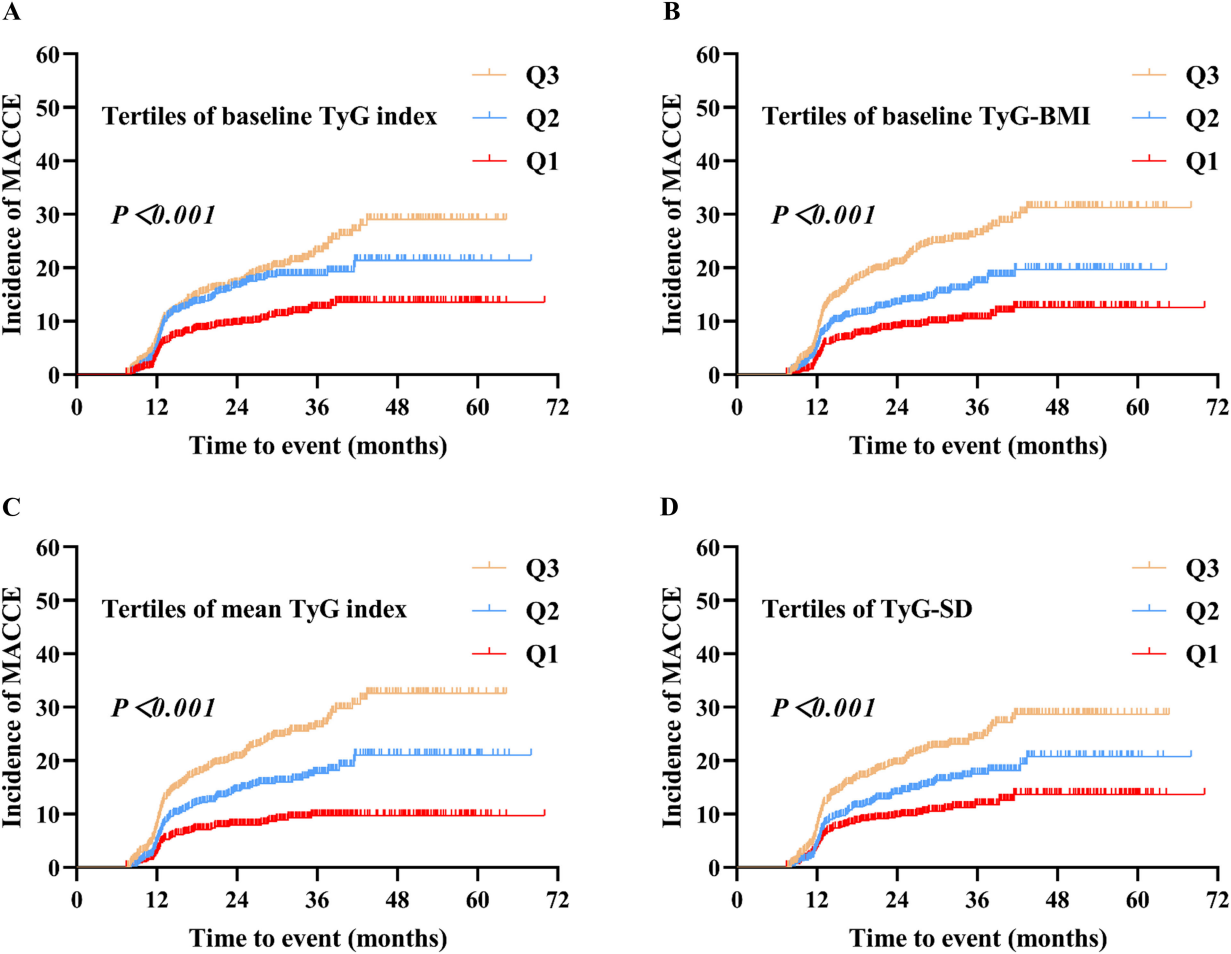
Kaplan–Meier analysis of MACCE by baseline TyG index **(A)**, baseline TyG-BMI **(B)**, mean TyG index **(C)**, and TyG-SD **(D)**. Four TyG indicators were grouped based on their tertiles, with the designation Q1 < Q2 < Q3. MACCE, major adverse cardiovascular and cerebrovascular events; TyG, triglyceride-glucose; TyG-BMI, triglyceride glucose-body mass index; TyG-SD, triglyceride glucose index-standard deviation.

The Kaplan-Meier survival analysis results indicated that when the four TyG indicators were stratified into tertiles, there were statistically significant differences in the cumulative incidence of MACCE among the three groups (P < 0.001). Furthermore, the cumulative incidence of MACCE increased with the higher TyG indicator tertiles (Q3 > Q2 > Q1) (**Figure 2**).

### Assessing predictive ability of the four TyG indicators for MACCE

**Figure 3:**
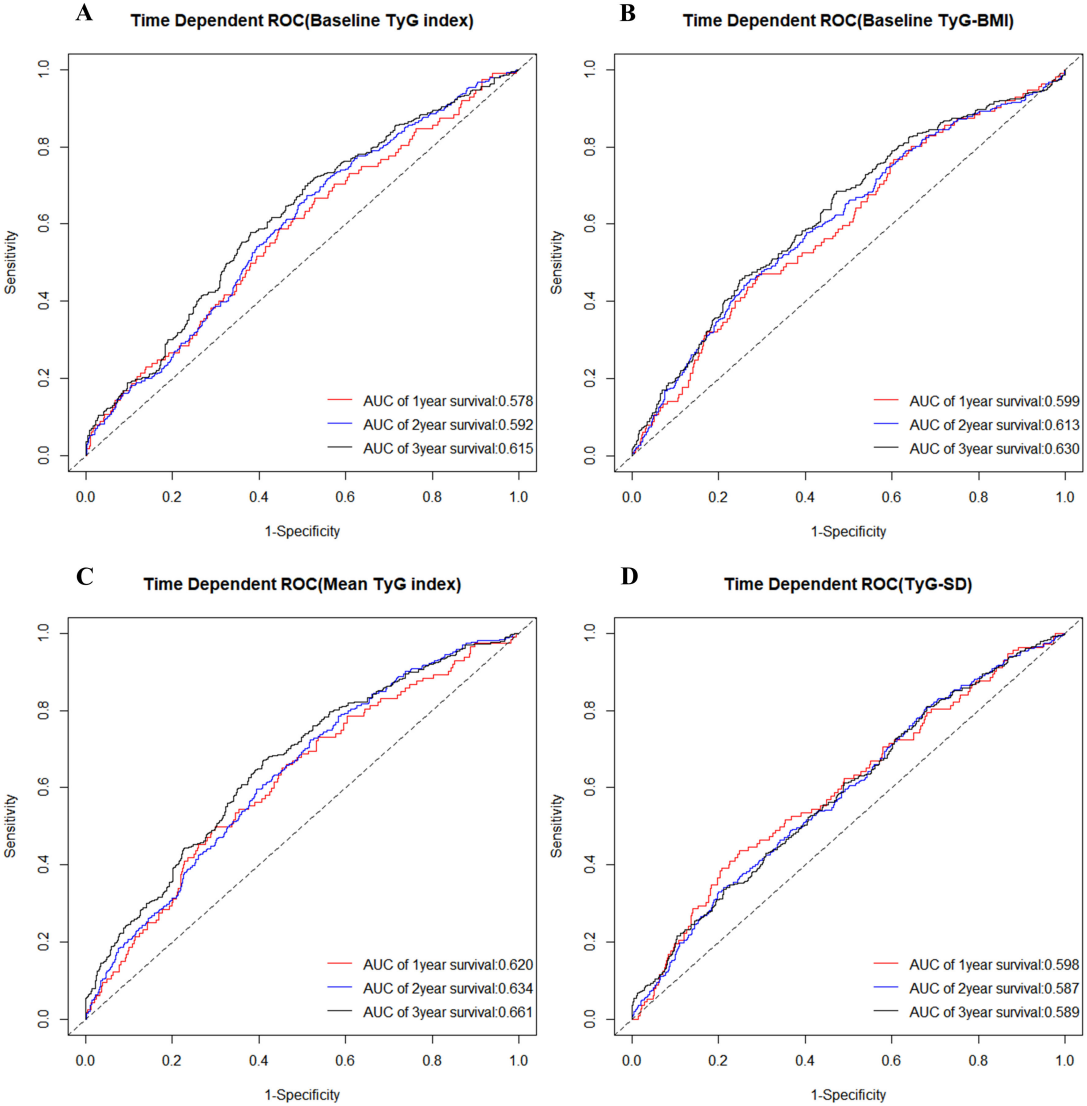
Time-dependent ROC curve for diagnosis of MACCE utilizing baseline TyG index **(A)**, baseline TyG-BMI **(B)**, mean TyG index **(C)**, and TyG-SD **(D)**. The AUC was calculated at the 1-year, 2-year, and 3-year time points, respectively. ROC, receiver operating characteristic; TyG, triglyceride-glucose; TyG-BMI, triglyceride glucose-body mass index; TyG-SD, triglyceride glucose index-standard deviation; AUC, area under curve; MACCE, major adverse cardiovascular and cerebrovascular events.

At different time points, **Figure 3** and **Table 3** presented the predictive ability of the four TyG indicators for MACCE in SCAD patients who underwent PCI, utilizing time-dependent ROC curves. Employing baseline TyG index as the detection variable, the area under curve (AUC) increased progressively over the three-year follow-up period, reaching 0.578 (95% CI: 0.524-0.633) in the first year, 0.592 (95% CI: 0.557-0.627) in the second year, and 0.615 (95% CI: 0.575-0.655) in the third year. Similarly, the predictive ability of baseline TyG-BMI and mean TyG index for MACCE also exhibited a steady increase over time. In contrast, when TyG-SD was the detection variable, the AUC was highest in the first year, followed by the third year, and lowest in the second year.

**Table 3:** The predictive value of four TyG indicators for MACCE at different time points.

Time points	Baseline TyG index	Baseline TyG-BMI	Mean TyG index	TyG-SD
AUC of 1 year	0.578 (0.524-0.633)	0.599 (0.545-0.653)	0.620 (0.567-0.672)	0.598 (0.543-0.653)
Cut-off of 1 year	8.52	231.45	8.65	0.3736
Youden index of 1 year	0.159	0.178	0.201	0.176
AUC of 2 year	0.592 (0.557-0.627)	0.613 (0.577-0.649)	0.634 (0.601-0.668)	0.587 (0.551-0.622)
Cut-off of 2 year	8.64	229.34	8.65	0.3722
Youden index of 2 year	0.171	0.184	0.208	0.164
AUC of 3 year	0.615 (0.575-0.655)	0.630 (0.592-0.669)	0.661 (0.623-0.700)	0.589 (0.549-0.629)
Cut-off of 3 year	8.65	232.18	8.66	0.3725
Youden index of 3 year	0.187	0.203	0.226	0.169

TyG, triglyceride-glucose; MACCE, major adverse cardiovascular and cerebrovascular events; AUC, area under curve; TyG-BMI, triglyceride glucose-body mass index; TyG-SD, triglyceride glucose index-standard deviation.

**Figure 4:**
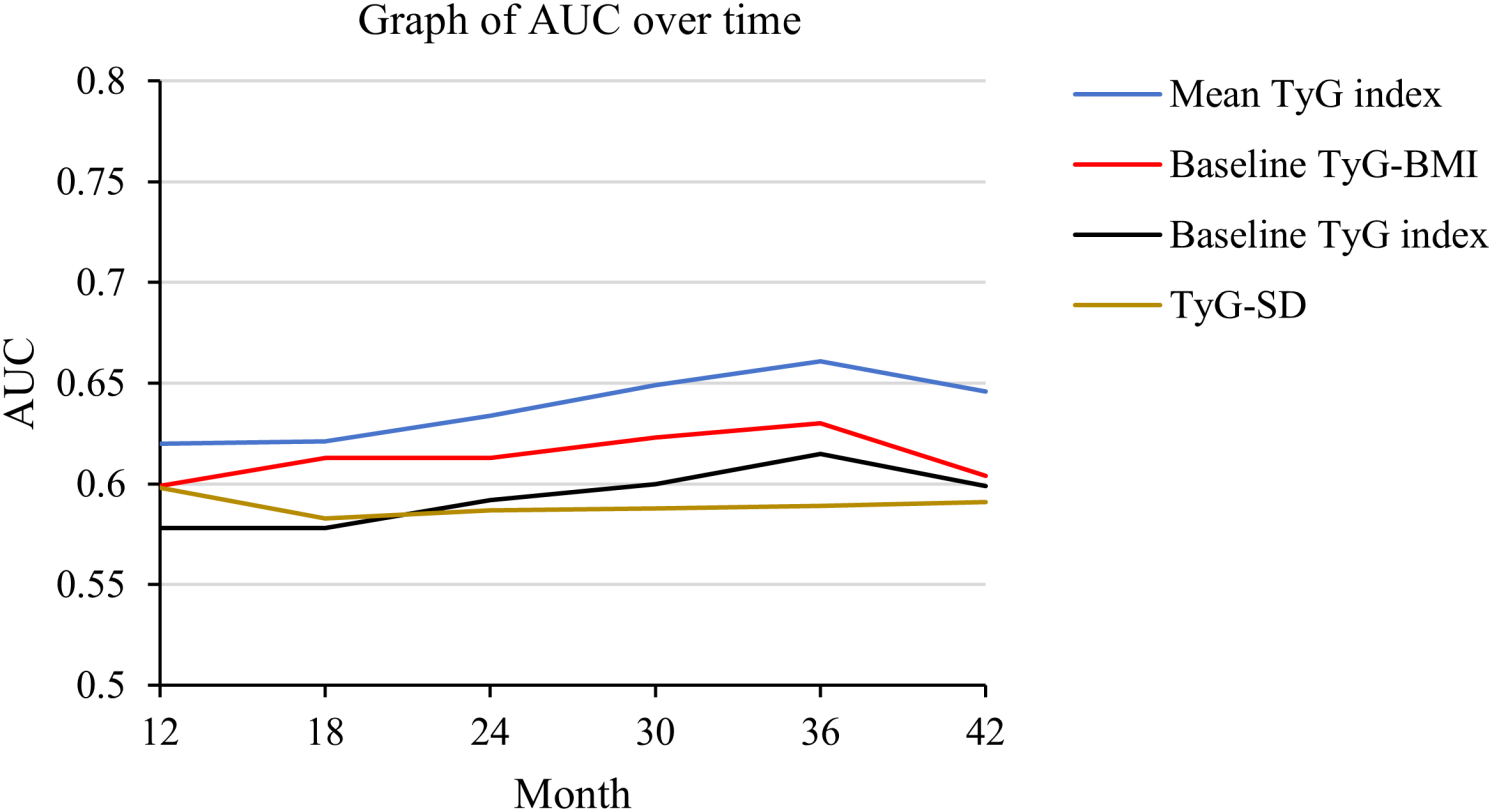
AUC changes over time (12, 18, 24, 30, 36, and 42 months) for the four TyG indicators in predicting MACCE. AUC, area under curve; TyG, triglyceride-glucose; TyG-BMI, triglyceride glucose-body mass index; TyG-SD, triglyceride glucose index-standard deviation; MACCE, major adverse cardiovascular and cerebrovascular events.

The study also compared the predictive ability of the four TyG indicators for MACCE across six time points (12, 18, 24, 30, 36, and 42 months). Among the four TyG indicators, regardless of the time point, the mean TyG index demonstrated the highest predictive ability for MACCE. Furthermore, at every time point, baseline TyG-BMI showed greater predictive accuracy for MACCE compared to baseline TyG index. For details, refer to **Figure 4** and **Table 4**.

**Table 4:** Comparison of the predictive value of four TyG indicators for MACCE across six time points.

Time points	Baseline TyG index	Baseline TyG-BMI	Mean TyG index	TyG-SD
AUC of 12 month	0.578 (0.524-0.633)	0.599 (0.545-0.653)	0.620 (0.567-0.672)	0.598 (0.543-0.653)
AUC of 18 month	0.578 (0.541-0.615)	0.613 (0.576-0.650)	0.621 (0.585-0.656)	0.583 (0.546-0.620)
AUC of 24 month	0.592 (0.557-0.627)	0.613 (0.577-0.649)	0.634 (0.601-0.668)	0.587 (0.551-0.622)
AUC of 30 month	0.600 (0.565-0.636)	0.623 (0.587-0.660)	0.649 (0.615-0.684)	0.588 (0.551-0.624)
AUC of 36 month	0.615 (0.575-0.655)	0.630 (0.592-0.669)	0.661 (0.623-0.700)	0.589 (0.549-0.629)
AUC of 42 month	0.599 (0.548-0.649)	0.604 (0.552-0.656)	0.646 (0.598-0.695)	0.591 (0.538-0.643)

TyG, triglyceride-glucose; MACCE, major adverse cardiovascular and cerebrovascular events; AUC, area under curve; TyG-BMI, triglyceride glucose-body mass index; TyG-SD, triglyceride glucose index-standard deviation.

### Subgroup analysis

This study also stratified individuals according to gender, diabetes, hypertension, and LDL-C levels to determine whether the association between the four TyG indicators and MACCE risk differed across various subgroups. The subgroup analysis found a significant positive association between the highest tertile levels of the four TyG indicators and MACCE risk (P < 0.05), irrespective of gender (**Supplementary Table 1**), diabetes status (**Supplementary Table 2**), or hypertension status (**Supplementary Table 3**). Similarly, in subgroups with LDL-C levels (< 1.8mmol/L or ≥ 1.8mmol/L) (**Supplementary Table 4**), a significant correlation was also found between the highest tertile levels of the four TyG indicators and MACCE risk (P < 0.05). Furthermore, within each subgroup, the analysis of the four TyG indicators as continuous variables also revealed a positive correlation with MACCE risk. These findings were consistent with the primary results observed in the overall population.

## Discussion

Based on a retrospective analysis of follow-up data from 2306 SCAD patients who underwent PCI, two main conclusions were drawn: 1. After thorough adjustment for covariates, higher levels of the four TyG indicators (baseline TyG index, baseline TyG-BMI, mean TyG index, TyG-SD) displayed an independent and positive correlation with MACCE risk. 2. Compared to baseline TyG index, baseline TyG-BMI, and TyG-SD, mean TyG index demonstrated superior predictive value for MACCE risk.

The TyG index has been established as an emerging indicator capable of representing IR, demonstrating a robust concordance with the outcomes of the gold-standard tests for IR (36, 37). Therefore, TyG index has initially shown substantial value in predicting metabolic diseases (38–40). Xue and colleagues found that TyG index, when combined with obesity indicators, could serve as an effective tool for the early screening of non-alcoholic fatty liver disease, metabolic-associated fatty liver disease, and liver fibrosis (38). Chen discovered that TyG index could identify normoglycemic conversion in prediabetes patients (39). Guo presented that TyG index was able to predict the onset of gestational diabetes in early pregnancy (40). Moreover, IR is known to facilitate the progression of CVD by inducing an imbalance in glucose metabolism, altering systemic lipid metabolism, and precipitating endothelial dysfunction (41, 42). Accordingly, some studies found that TyG index also demonstrated high accuracy in predicting the onset and prognosis of CVD (43–45). Zhao showed that TyG index was positively correlated with chest pain events and all-cause mortality in United States adults (8). Xin proposed that both baseline TyG index and the elevated trajectory of its growth were linked to hypertension incidence (46). Wu et al. suggested that among patients over 45 years old with consistently elevated TyG index levels, the incidence of stroke was significantly higher (47). Zhou indicated that TyG index was strongly correlated with a higher risk of both mortality and rehospitalization in patients with heart failure with preserved ejection fraction (48). Guo et al. also identified that people with elevated TyG index values had a greater probability of experiencing impaired cardiovascular fitness, particularly among males (49). Santulli reported that prediabetes may increase the risk of frailty in elderly individuals with hypertension, with the TyG index potentially serving as an intermediary in this association (50). However, the aforementioned studies have seldom addressed the relationship between TyG index and prognosis in SCAD patients. Given that SCAD is a critical component of CVD, it deserves greater attention. Therefore, this study aimed to fill this void.

This study also combined TyG index with BMI and considered the longitudinal patterns of TyG index (mean level and variability). Hence, it adopted a multidimensional approach to examine the relationship between TyG index and the prognosis in SCAD patients. Similar to previous studies (11, 21, 32, 51), this study demonstrated that baseline TyG index, baseline TyG-BMI, mean TyG index, and TyG-SD were all significantly associated with adverse prognosis in SCAD patients, with these findings remaining robust across multiple subgroups. Interestingly, our study also revealed that, at several time points, mean TyG index exhibited the highest predictive value for adverse outcomes, while baseline TyG-BMI showed greater predictive ability than baseline TyG index. This may be attributable to the fact that mean TyG index and TyG-BMI better reflect the real status of IR (17, 46), suggesting that clinicians may need to pay more attention to populations with persistently high TyG index levels over the long term. Furthermore, in situations where only baseline data were available, prioritizing the assessment of TyG-BMI would be a more valuable approach.

Prior studies indicated that the variability of certain risk factors for CVD, such as blood pressure, glucose, and lipid, could predict adverse outcomes in CVD patients, with this predictive effect being independent of the baseline or mean levels of the risk factors (52–54). Our study also found that the variability of TyG index was associated with adverse prognosis in SCAD patients. Several potential pathophysiological mechanisms might account for the results of this study. Initially, TyG index was correlated with FBG, and drastic fluctuations in FBG could induce oxidative stress, elevate levels of inflammatory cytokines, and cause endothelial dysfunction. This process may accelerate the occurrence of atherosclerosis, thereby triggering the onset of adverse events (55, 56). Secondly, TyG index serves as a biomarker for IR levels, the fluctuations of which may precipitate alterations in lipid levels. The high variability of lipids could potentially induce the crystallization and dissolution of cholesterol within the coronary artery plaques, thereby augmenting the risk of plaque rupture, which implicates a critical pathophysiological process implicated in AMI and stroke (57, 58).

### Study strengths and limitations

This study was the first to investigate the relationship between TyG-related indicators and clinical prognosis in SCAD patients undergoing PCI from multiple perspectives, and to compare the predictive efficacy of the four TyG indicators (baseline TyG index, baseline TyG-BMI, mean TyG index, and TyG-SD) for adverse outcomes at various time points. It offered new insights for clinicians in identifying individuals at high risk for MACCE. However, the study also has several limitations. Firstly, given the observational nature of this study, we were unable to establish a causal relationship between the four TyG indicators and MACCE risk. Secondly, despite statistical adjustments for confounding variables, it was not possible to entirely rule out biases and confounding factors. Additionally, this study involved a relatively small sample size. Consequently, the conclusions drawn from the current study require further validation through larger-scale prospective studies. Fourthly, the individuals of this study were selected from the Chinese population, and further research may be needed to validate the applicability of these findings to other racial groups. Moreover, as only participants with a minimum of two TyG index assessments during the first two years post-PCI were included, and the frequency of assessments varied among patients, there may be a certain degree of selection bias in this study. Then, the study only included SCAD patients who underwent PCI, excluding those managed with drug therapy alone. Therefore, the current results were unable to generalized to all SCAD patients. Lastly, given the relatively short median follow-up duration of about 26.1 months, the results should ideally be corroborated by future research encompassing longer follow-up periods.

## Conclusion

Overall, this study expanded the use of the TyG index and its derived indicators to SCAD patients undergoing PCI, demonstrating that higher levels of baseline TyG index, baseline TyG-BMI, mean TyG index, and TyG index variability were independently positively associated with MACCE risk in these patients. Among these, mean TyG index may serve as a more reliable tool for identifying individuals at higher risk of CVD events. Thus, long-term tracking of TyG index in clinical practice warrants considerable attention.

## Data Availability

The original contributions presented in the study are included in the article/**Supplementary Material**. Further inquiries can be directed to the corresponding author.

## References

[1] Feng X Navakatikyan M Eckermann S Astell-Burt T. Show me the money! Associations between tree canopy and hospital costs in cities for cardiovascular disease events in a longitudinal cohort study of 110,134 participants. Environ Int. (2024) 185:108558. doi: 10.1016/j.envint.2024.108558 38490071

[2] Birger M Kaldjian AS Roth GA Moran AE Dieleman JL Bellows BK. Spending on cardiovascular disease and cardiovascular risk factors in the United States: 1996 to 2016. Circulation. (2021) 144:271–82. doi: 10.1161/CIRCULATIONAHA.120.053216 PMC8316421 33926203

[3] Louie JZ Shiffman D McPhaul MJ Melander O. Insulin resistance probability score and incident cardiovascular disease. J Internal Med. (2023) 294:531–5. doi: 10.1111/joim.v294.4 37424183

[4] Hill MA Yang Y Zhang L Sun Z Jia G Parrish AR. Insulin resistance, cardiovascular stiffening and cardiovascular disease. Metabolism: Clin experimental. (2021) 119:154766. doi: 10.1016/j.metabol.2021.154766 33766485

[5] Oliveri A Rebernick RJ Kuppa A Pant A Chen Y Du X. Comprehensive genetic study of the insulin resistance marker TG : HDL-C in the UK Biobank. Nat Genet. (2024) 56:212–21. doi: 10.1038/s41588-023-01625-2 PMC10923176 38200128

[6] Cui C Liu L Qi Y Han N Xu H Wang Z. Joint association of TyG index and high sensitivity C-reactive protein with cardiovascular disease: a national cohort study. Cardiovasc diabetology. (2024) 23:156. doi: 10.1186/s12933-024-02244-9 PMC11077847 38715129

[7] Liu C Liang D Xiao K Xie L. Association between the triglyceride-glucose index and all-cause and CVD mortality in the young population with diabetes. Cardiovasc diabetology. (2024) 23:171. doi: 10.1186/s12933-024-02269-0 PMC11097545 38755682

[8] Zhao Y Gu Y Zhang B. Associations of triglyceride-glucose (TyG) index with chest pain incidence and mortality among the U.S. population. Cardiovasc Diabetol. (2024) 23:111. doi: 10.1186/s12933-024-02209-y 38555461 PMC10981836

[9] Zheng D Cai J Xu S Jiang S Li C Wang B. The association of triglyceride-glucose index and combined obesity indicators with chest pain and risk of cardiovascular disease in American population with pre-diabetes or diabetes. Front endocrinology. (2024) 15:1471535. doi: 10.3389/fendo.2024.1471535 PMC11412814 39309107

[10] Zhang Y Wang F Tang J Shen L He J Chen Y. Association of triglyceride glucose-related parameters with all-cause mortality and cardiovascular disease in NAFLD patients: NHANES 1999-2018. Cardiovasc diabetology. (2024) 23:262. doi: 10.1186/s12933-024-02354-4 PMC11264797 39026233

[11] Cui C Liu L Zhang T Fang L Mo Z Qi Y. Triglyceride-glucose index, renal function and cardiovascular disease: a national cohort study. Cardiovasc diabetology. (2023) 22:325. doi: 10.1186/s12933-023-02055-4 PMC10685637 38017519

[12] Molavizadeh D Cheraghloo N Tohidi M Azizi F Hadaegh F. The association between index-year, average, and variability of the triglyceride-glucose index with health outcomes: more than a decade of follow-up in Tehran lipid and glucose study. Cardiovasc diabetology. (2024) 23:321. doi: 10.1186/s12933-024-02387-9 PMC11365227 39217401

[13] Wang Z He H Xie Y Li J Luo F Sun Z. Non-insulin-based insulin resistance indexes in predicting atrial fibrillation recurrence following ablation: a retrospective study. Cardiovasc diabetology. (2024) 23:87. doi: 10.1186/s12933-024-02158-6 PMC10902970 38419016

[14] Xia X Chen S Tian X Xu Q Zhang Y Zhang X. Association of triglyceride-glucose index and its related parameters with atherosclerotic cardiovascular disease: evidence from a 15-year follow-up of Kailuan cohort. Cardiovasc diabetology. (2024) 23:208. doi: 10.1186/s12933-024-02290-3 PMC11188278 38898520

[15] Koskinas KC Van Craenenbroeck EM Antoniades C Blüher M Gorter TM Hanssen H. Obesity and cardiovascular disease: an ESC clinical consensus statement. Eur Heart J. (2024) 45:4063–98. doi: 10.1093/eurheartj/ehae508 39210706

[16] Dang K Wang X Hu J Zhang Y Cheng L Qi X. The association between triglyceride-glucose index and its combination with obesity indicators and cardiovascular disease: NHANES 2003-2018. Cardiovasc diabetology. (2024) 23:8. doi: 10.1186/s12933-023-02115-9 PMC10771672 38184598

[17] Zhang Y Wang R Fu X Song H. Non-insulin-based insulin resistance indexes in predicting severity for coronary artery disease. Diabetol Metab syndrome. (2022) 14:191. doi: 10.1186/s13098-022-00967-x PMC9759860 36528713

[18] Hou Q Zhang H Zhang R Li B Li L Li D. Relationship between the longitudinal trajectory of the triglyceride-glucose index and the development of CKD: an 8-year retrospective longitudinal cohort study. Front endocrinology. (2024) 15:1376166. doi: 10.3389/fendo.2024.1376166 PMC11163917 38859908

[19] Chen N Ma LL Zhang Y Chu X Dong J Yan YX. Association of long-term triglyceride-glucose index patterns with the incidence of chronic kidney disease among non-diabetic population: evidence from a functional community cohort. Cardiovasc diabetology. (2024) 23:7. doi: 10.1186/s12933-023-02098-7 PMC10765660 38172903

[20] Zhu X Xu W Song T Wang X Wang Q Li J. Changes in the combination of the triglyceride-glucose index and obesity indicators estimate the risk of cardiovascular disease. Cardiovasc diabetology. (2024) 23:192. doi: 10.1186/s12933-024-02281-4 PMC11157789 38844974

[21] Li H Zuo Y Qian F Chen S Tian X Wang P. Triglyceride-glucose index variability and incident cardiovascular disease: a prospective cohort study. Cardiovasc diabetology. (2022) 21:105. doi: 10.1186/s12933-022-01541-5 PMC9188105 35689232

[22] Wang C Liao P Tang C Chen C Zhang X. The predictive value of the triglyceride glucose index combined with cystatin C for the prognosis of patients with acute coronary syndrome. Front endocrinology. (2024) 15:1423227. doi: 10.3389/fendo.2024.1423227 PMC11385367 39257901

[23] Liu H Wang L Wang H Hao X Du Z Li C. The association of triglyceride-glucose index with major adverse cardiovascular and cerebrovascular events after acute myocardial infarction: a meta-analysis of cohort studies. Nutr diabetes. (2024) 14:39. doi: 10.1038/s41387-024-00295-1 38844442 PMC11156940

[24] Khalaji A Behnoush AH Pasebani Y Rafati A Mahmoodi T Arzhangzadeh A. Triglyceride-glucose index as a predictor of cardiac adverse events in acute coronary syndrome patients undergoing percutaneous coronary intervention: role of diabetes. BMC Cardiovasc Disord. (2024) 24:514. doi: 10.1186/s12872-024-04191-5 39333881 PMC11430238

[25] Chen Q Xiong S Ye T Gao Y Wang J Li X. Insulin resistance, coronary artery lesion complexity and adverse cardiovascular outcomes in patients with acute coronary syndrome. Cardiovasc diabetology. (2024) 23:172. doi: 10.1186/s12933-024-02276-1 PMC11100181 38755609

[26] Montalescot G Sechtem U Achenbach S Andreotti F Arden C Budaj A. 2013 ESC guidelines on the management of stable coronary artery disease: the Task Force on the management of stable coronary artery disease of the European Society of Cardiology. Eur Heart J. (2013) 34:2949–3003. doi: 10.1093/eurheartj/eht296 23996286

[27] Kornowski R Mehran R Dangas G Nikolsky E Assali A Claessen BE. Prognostic impact of staged versus "one-time" multivessel percutaneous intervention in acute myocardial infarction: analysis from the HORIZONS-AMI (harmonizing outcomes with revascularization and stents in acute myocardial infarction) trial. J Am Coll Cardiol. (2011) 58:704–11. doi: 10.1016/j.jacc.2011.02.071 21816305

[28] Gou R Dou D Tian M Chang X Zhao Y Meng X. Association between triglyceride glucose index and hyperuricemia: a new evidence from China and the United States. Front endocrinology. (2024) 15:1403858. doi: 10.3389/fendo.2024.1403858 PMC11246899 39010899

[29] Ma YL Tang XF Yao Y Xu N Song Y Jiang P. Comparison of efficacy and safety between first- and second-generation drug-eluting stents in patients with acute coronary syndrome. Chin Med J. (2018) 131:1397–405. doi: 10.4103/0366-6999.233959 PMC6006822 29893356

[30] Cheng Y Fang Z Zhang X Wen Y Lu J He S. Association between triglyceride glucose-body mass index and cardiovascular outcomes in patients undergoing percutaneous coronary intervention: a retrospective study. Cardiovasc diabetology. (2023) 22:75. doi: 10.1186/s12933-023-01794-8 PMC10064664 36997935

[31] Song Y Cui K Yang M Song C Yin D Dong Q. High triglyceride-glucose index and stress hyperglycemia ratio as predictors of adverse cardiac events in patients with coronary chronic total occlusion: a large-scale prospective cohort study. Cardiovasc diabetology. (2023) 22:180. doi: 10.1186/s12933-023-01883-8 PMC10350280 37454147

[32] Wang Y Wang Y Sun S Liu X Zhao W Li W. Triglyceride-glucose index level and variability and outcomes in patients with acute coronary syndrome undergoing percutaneous coronary intervention: an observational cohort study. Lipids Health disease. (2022) 21:134. doi: 10.1186/s12944-022-01731-w PMC9733246 36482415

[33] Zhao Q Cheng YJ Xu YK Zhao ZW Liu C Sun TN. Comparison of various insulin resistance surrogates on prognostic prediction and stratification following percutaneous coronary intervention in patients with and without type 2 diabetes mellitus. Cardiovasc diabetology. (2021) 20:190. doi: 10.1186/s12933-021-01383-7 PMC8449896 34537077

[34] Hosseini Mojahed F Aalami AH Pouresmaeil V Amirabadi A Qasemi Rad M Sahebkar A. Clinical evaluation of the diagnostic role of microRNA-155 in breast cancer. Int J Genomics. (2020) 2020:9514831. doi: 10.1155/2020/9514831 32964011 PMC7495225

[35] Santulli G Pascale V Finelli R Visco V Giannotti R Massari A. We are what we eat: impact of food from short supply chain on metabolic syndrome. J Clin Med. (2019) 8. doi: 10.3390/jcm8122061 PMC6947359 31771147

[36] Mohd Nor NS Lee S Bacha F Tfayli H Arslanian S. Triglyceride glucose index as a surrogate measure of insulin sensitivity in obese adolescents with normoglycemia, prediabetes, and type 2 diabetes mellitus: comparison with the hyperinsulinemic-euglycemic clamp. Pediatr diabetes. (2016) 17:458–65. doi: 10.1111/pedi.2016.17.issue-6 26251318

[37] Tao LC Xu JN Wang TT Hua F Li JJ. Triglyceride-glucose index as a marker in cardiovascular diseases: landscape and limitations. Cardiovasc diabetology. (2022) 21:68. doi: 10.1186/s12933-022-01511-x PMC9078015 35524263

[38] Xue Y Xu J Li M Gao Y. Potential screening indicators for early diagnosis of NAFLD/MAFLD and liver fibrosis: Triglyceride glucose index-related parameters. Front endocrinology. (2022) 13:951689. doi: 10.3389/fendo.2022.951689 PMC9478620 36120429

[39] Chen X Liu D He W Hu H Wang W. Predictive performance of triglyceride glucose index (TyG index) to identify glucose status conversion: a 5-year longitudinal cohort study in Chinese pre-diabetes people. J Trans Med. (2023) 21:624. doi: 10.1186/s12967-023-04402-1 PMC10503019 37715242

[40] Guo Y Lu J Bahani M Ding G Wang L Zhang Y. Triglyceride-glucose index in early pregnancy predicts the risk of gestational diabetes: a prospective cohort study. Lipids Health disease. (2024) 23:87. doi: 10.1186/s12944-024-02076-2 PMC10962154 38528508

[41] Beverly JK Budoff MJ. Atherosclerosis: Pathophysiology of insulin resistance, hyperglycemia, hyperlipidemia, and inflammation. J diabetes. (2020) 12:102–4. doi: 10.1111/1753-0407.12970 31411812

[42] Martyn JA Kaneki M Yasuhara S. Obesity-induced insulin resistance and hyperglycemia: etiologic factors and molecular mechanisms. Anesthesiology. (2008) 109:137–48. doi: 10.1097/ALN.0b013e3181799d45 PMC3896971 18580184

[43] Liu X Tan Z Huang Y Zhao H Liu M Yu P. Relationship between the triglyceride-glucose index and risk of cardiovascular diseases and mortality in the general population: a systematic review and meta-analysis. Cardiovasc diabetology. (2022) 21:124. doi: 10.1186/s12933-022-01546-0 PMC9250255 35778731

[44] Xu X Huang R Lin Y Guo Y Xiong Z Zhong X. High triglyceride-glucose index in young adulthood is associated with incident cardiovascular disease and mortality in later life: insight from the CARDIA study. Cardiovasc diabetology. (2022) 21:155. doi: 10.1186/s12933-022-01593-7 PMC9375240 35962377

[45] Yu Y Gu M Huang H Cheng S Deng Y Cai C. Combined association of triglyceride-glucose index and systolic blood pressure with all-cause and cardiovascular mortality among the general population. J Trans Med. (2022) 20:478. doi: 10.1186/s12967-022-03678-z PMC9583494 36266665

[46] Xin F He S Zhou Y Jia X Zhao Y Zhao H. The triglyceride glucose index trajectory is associated with hypertension: a retrospective longitudinal cohort study. Cardiovasc diabetology. (2023) 22:347. doi: 10.1186/s12933-023-02087-w PMC10725029 38102704

[47] Wu Y Yang Y Zhang J Liu S Zhuang W. The change of triglyceride-glucose index may predict incidence of stroke in the general population over 45 years old. Cardiovasc diabetology. (2023) 22:132. doi: 10.1186/s12933-023-01870-z PMC10257314 37296457

[48] Zhou Q Yang J Tang H Guo Z Dong W Wang Y. High triglyceride-glucose (TyG) index is associated with poor prognosis of heart failure with preserved ejection fraction. Cardiovasc diabetology. (2023) 22:263. doi: 10.1186/s12933-023-02001-4 PMC10541699 37775762

[49] Guo D Wu Z Xue F Chen S Ran X Zhang C. Association between the triglyceride-glucose index and impaired cardiovascular fitness in non-diabetic young population. Cardiovasc diabetology. (2024) 23:39. doi: 10.1186/s12933-023-02089-8 PMC10800072 38245734

[50] Santulli G Visco V Varzideh F Guerra G Kansakar U Gasperi M. Prediabetes increases the risk of frailty in prefrail older adults with hypertension: beneficial effects of metformin. Hypertension (Dallas Tex: 1979). (2024) 81:1637–43. doi: 10.1161/HYPERTENSIONAHA.124.23087 PMC11170724 38752357

[51] Jin JL Sun D Cao YX Guo YL Wu NQ Zhu CG. Triglyceride glucose and haemoglobin glycation index for predicting outcomes in diabetes patients with new-onset, stable coronary artery disease: a nested case-control study. Ann Med. (2018) 50:576–86. doi: 10.1080/07853890.2018.1523549 30207490

[52] Echouffo-Tcheugui JB Zhao S Brock G Matsouaka RA Kline D Joseph JJ. Visit-to-visit glycemic variability and risks of cardiovascular events and all-cause mortality: the ALLHAT study. Diabetes Care. (2019) 42:486–93. doi: 10.2337/dc18-1430 PMC6463548 30659073

[53] Stevens SL Wood S Koshiaris C Law K Glasziou P Stevens RJ. Blood pressure variability and cardiovascular disease: systematic review and meta-analysis. BMJ (Clinical Res ed). (2016) 354:i4098. doi: 10.1136/bmj.i4098 PMC4979357 27511067

[54] Wan EYF Yu EYT Chin WY Barrett JK Mok AHY Lau CST. Greater variability in lipid measurements associated with cardiovascular disease and mortality: A 10-year diabetes cohort study. Diabetes Obes Metab. (2020) 22:1777–88. doi: 10.1111/dom.v22.10 PMC7540339 32452623

[55] An Y Xu BT Wan SR Ma XM Long Y Xu Y. The role of oxidative stress in diabetes mellitus-induced vascular endothelial dysfunction. Cardiovasc diabetology. (2023) 22:237. doi: 10.1186/s12933-023-01965-7 PMC10475205 37660030

[56] Maruhashi T Higashi Y. Pathophysiological association between diabetes mellitus and endothelial dysfunction. Antioxidants (Basel Switzerland). (2021) 10. doi: 10.3390/antiox10081306 PMC8389282 34439553

[57] Choi SH Ginsberg HN. Increased very low density lipoprotein (VLDL) secretion, hepatic steatosis, and insulin resistance. Trends Endocrinol metabolism: TEM. (2011) 22:353–63. doi: 10.1016/j.tem.2011.04.007 PMC3163828 21616678

[58] Nagle CA Klett EL Coleman RA. Hepatic triacylglycerol accumulation and insulin resistance. J Lipid Res. (2009) 50 Suppl:S74–9. doi: 10.1194/jlr.R800053-JLR200 PMC2674743 18997164

