# Supplementary material for: The prognostic association of triglyceride-glucose index and its derived indicators with stable coronary artery disease patients undergoing percutaneous coronary intervention
Source: Front Endocrinol (Lausanne). 2025 Jan 22;16:1465376. doi: 10.3389/fendo.2025.1465376 (PMC11794097; doi:10.3389/fendo.2025.1465376)
Supplement: Supplementary file 1 [file Table1.docx]

The prognostic association of triglyceride-glucose index and its derived indicators with stable coronary artery disease patients undergoing percutaneous coronary intervention

Yu Shan, MD^1,2†^, Maoning Lin, MD^1,2†^, Fangfang Gu, MD^3^, Duanbin Li, MD^1,2^, Qiongjun Zhu, MD^1,2^, Zhezhe Chen, MD^1,2^, Wenbin Zhang, MD, PHD,^1,2^, Guosheng Fu, MD, PHD, ^1,2*^, Min Wang, MD, PHD, ^1,2*^

^1^Department of Cardiology, Sir Run Run Shaw Hospital, College of Medicine, Zhejiang University, No 3 East of Qinchun Road, Hangzhou, Zhejiang 310000, China

^2^Key Laboratory of Cardiovascular Intervention and Regenerative Medicine of Zhejiang Province, Hangzhou, China

^3^Department of Cardiology, The Affiliated Huzhou Hospital (Huzhou Central Hospital), College of Medicine, Zhejiang University, NO 1558 North Sanhuan Road, Huzhou, Zhejiang 313000, China

† Yu Shan and Maoning Lin have contributed equally to this work and share the first authorship.

* Min Wang and Guosheng Fu are the corresponding authors of this article.

*** Correspondence:**Min Wang, MD PhD

Address: Department of Cardiology, Sir Run Run Shaw Hospital, College of Medicine, Zhejiang University, No 3 East of Qinchun Road, Hangzhou, Zhejiang 310000, China; Key Laboratory of Cardiovascular Intervention and Regenerative Medicine of Zhejiang Province, Hangzhou, China.

E-mail: wangminsyf30508@zju.edu.cn

Keywords: Insulin resistance; Triglyceride-glucose index; Percutaneous coronary intervention; Stable coronary artery disease; Major adverse cardiovascular and cerebrovascular events.

**Supplementary Material**

**Supplementary Table 1.** Subgroup analysis according to stratification of gender. **P3**

**Supplementary Table 2.** Subgroup analysis according to stratification of diabetes. **P5**

**Supplementary Table 3.** Subgroup analysis according to stratification of hypertension. **P7**

**Supplementary Table 4.** Subgroup analysis according to stratification of LDL-C levels. **P9**

**Supplementary Table 1** Subgroup analysis according to stratification of gender

| Variables | Model 1 |  |  | Model 2 |  |  | Model 3 |  |
| --- | --- | --- | --- | --- | --- | --- | --- | --- |
|  | Adjusted HR (95%CI) | *P* value |  | Adjusted HR (95%CI) | *P* value |  | Adjusted HR (95%CI) | *P* value |
| **Female** |  |  |  |  |  |  |  |  |
| Baseline TyG index | 2.170 (1.701-2.768) | <0.001* |  | 1.710 (1.292-2.264) | <0.001* |  | 1.757 (1.326-2.327) | <0.001* |
| Q1 | 1 (Reference) |  |  | 1 (Reference) |  |  | 1 (Reference) |  |
| Q2 | 1.866 (1.010-3.445) | 0.046* |  | 1.596 (0.858-2.970) | 0.140 |  | 1.681 (0.900-3.141) | 0.103 |
| Q3 | 3.326 (1.884-5.874) | <0.001* |  | 2.266 (1.252-4.099) | 0.007* |  | 2.308 (1.261-4.224) | 0.007* |
|  |  |  |  |  |  |  |  |  |
| Baseline TyG-BMI | 1.013 (1.009-1.017) | <0.001* |  | 1.010 (1.006-1.015) | <0.001* |  | 1.011 (1.006-1.015) | <0.001* |
| Q1 | 1 (Reference) |  |  | 1 (Reference) |  |  | 1 (Reference) |  |
| Q2 | 1.981 (1.124-3.489) | 0.018* |  | 1.811 (1.026-3.199) | 0.041* |  | 1.741 (0.980-3.091) | 0.059 |
| Q3 | 3.903 (2.341-6.507) | <0.001* |  | 3.224 (1.914-5.431) | <0.001* |  | 3.329 (1.965-5.642) | <0.001* |
|  |  |  |  |  |  |  |  |  |
| Mean TyG index | 2.942 (2.213-3.913) | <0.001* |  | 2.204 (1.574-3.088) | <0.001* |  | 2.402 (1.679-3.438) | <0.001* |
| Q1 | 1 (Reference) |  |  | 1 (Reference) |  |  | 1 (Reference) |  |
| Q2 | 3.648 (1.614-8.249) | 0.002* |  | 3.349 (1.473-7.614) | 0.004* |  | 3.404 (1.495-7.748) | 0.004* |
| Q3 | 6.696 (3.071-14.601) | <0.001* |  | 4.719 (2.115-10.530) | <0.001* |  | 4.635 (2.067-10.397) | <0.001* |
|  |  |  |  |  |  |  |  |  |
| TyG-SD | 4.961 (2.466-9.980) | <0.001* |  | 3.167 (1.417-7.079) | 0.005* |  | 2.878 (1.271-6.515) | 0.011* |
| Q1 | 1 (Reference) |  |  | 1 (Reference) |  |  | 1 (Reference) |  |
| Q2 | 2.373 (1.343-4.195) | 0.003* |  | 2.252 (1.269-3.993) | 0.006* |  | 2.281 (1.279-4.068) | 0.005* |
| Q3 | 2.879 (1.660-4.991) | <0.001* |  | 2.305 (1.305-4.071) | 0.004* |  | 2.258 (1.267-4.025) | 0.006* |
|  |  |  |  |  |  |  |  |  |
| **Male** |  |  |  |  |  |  |  |  |
| Baseline TyG index | 1.684 (1.405-2.019) | <0.001* |  | 1.479 (1.216-1.799) | <0.001* |  | 1.384 (1.125-1.702) | 0.002* |
| Q1 | 1 (Reference) |  |  | 1 (Reference) |  |  | 1 (Reference) |  |
| Q2 | 1.669 (1.202-2.317) | 0.002* |  | 1.473 (1.054-2.057) | 0.023* |  | 1.397 (0.997-1.957) | 0.052 |
| Q3 | 2.057 (1.494-2.833) | <0.001* |  | 1.658 (1.186-2.319) | 0.003* |  | 1.458 (1.031-2.062) | 0.033* |
|  |  |  |  |  |  |  |  |  |
| Baseline TyG-BMI | 1.011 (1.007-1.014) | <0.001* |  | 1.010 (1.006-1.013) | <0.001* |  | 1.009 (1.005-1.013) | <0.001* |
| Q1 | 1 (Reference) |  |  | 1 (Reference) |  |  | 1 (Reference) |  |
| Q2 | 1.587 (1.110-2.267) | 0.011* |  | 1.500 (1.046-2.151) | 0.027* |  | 1.497 (1.044-2.148) | 0.028* |
| Q3 | 2.627 (1.875-3.682) | <0.001* |  | 2.362 (1.670-3.341) | <0.001* |  | 2.251 (1.585-3.196) | <0.001* |
|  |  |  |  |  |  |  |  |  |
| Mean TyG index | 2.420 (1.939-3.020) | <0.001* |  | 2.110 (1.652-2.696) | <0.001* |  | 2.040 (1.588-2.620) | <0.001* |
| Q1 | 1 (Reference) |  |  | 1 (Reference) |  |  | 1 (Reference) |  |
| Q2 | 1.787 (1.266-2.523) | 0.001* |  | 1.627 (1.147-2.308) | 0.006* |  | 1.551 (1.091-2.207) | 0.015* |
| Q3 | 2.827 (2.037-3.924) | <0.001* |  | 2.259 (1.592-3.203) | <0.001* |  | 2.161 (1.518-3.076) | <0.001* |
|  |  |  |  |  |  |  |  |  |
| TyG-SD | 2.727 (1.982-3.753) | <0.001* |  | 2.316 (1.616-3.317) | <0.001* |  | 2.421 (1.656-3.541) | <0.001* |
| Q1 | 1 (Reference) |  |  | 1 (Reference) |  |  | 1 (Reference) |  |
| Q2 | 1.281 (0.905-1.812) | 0.162 |  | 1.189 (0.839-1.684) | 0.331 |  | 1.179 (0.831-1.674) | 0.356 |
| Q3 | 2.115 (1.542-2.901) | <0.001* |  | 1.824 (1.318-2.523) | <0.001* |  | 1.779 (1.282-2.468) | 0.001* |

**Model 1**: Adjusted for age and gender (male or female).

**Model 2**: Adjusted for age, gender (male or female), baseline BMI, smoke (never, former, and current), drink (never, former, and current), hypertension (yes or no), and diabetes (yes or no).

**Model 3**: Adjusted for age, gender (male or female), baseline BMI, smoke (never, former, and current), drink (never, former, and current), hypertension (yes or no), and diabetes (yes or no), prior AMI (yes or no), baseline LDL-C, multivessel lesion (yes or no), LVEF, and medications (administration of ACEI/ARB, antidiabetic agents, antihypertensive agents, and β-receptor blocker) (yes or no).

The four TyG indicators were analyzed as continuous variables in Cox regression models. Moreover, the study also stratified participants into tertiles based on four TyG indicators (baseline TyG index, baseline TyG-BMI, mean TyG index, and TyG-SD), with the designation Q1<Q2<Q3, using Q1 as the reference group. Given the significant collinearity observed between the TyG-BMI and the BMI, the model employed at baseline TyG-BMI excludes the BMI as a variable. TyG: triglyceride-glucose; TyG-BMI: triglyceride glucose-body mass index; TyG-SD: triglyceride glucose index-standard deviation; HR: hazard ratios; CI: confidence interval; BMI: body mass index; AMI: acute myocardial infarction; LDL-C: low density lipoprotein cholesterol; LVEF: left ventricular ejection fraction; ACEI: angiotensin converting enzyme inhibitor; ARB: angiotensin receptor blocker; *: P < 0.05.

**Supplementary Table 2** Subgroup analysis according to stratification of diabetes

| Variables | Model 1 |  |  | Model 2 |  |  | Model 3 |  |
| --- | --- | --- | --- | --- | --- | --- | --- | --- |
|  | Adjusted HR (95%CI) | *P* value |  | Adjusted HR (95%CI) | *P* value |  | Adjusted HR (95%CI) | *P* value |
| **No diabetes** |  |  |  |  |  |  |  |  |
| Baseline TyG index | 1.598 (1.280-1.995) | <0.001* |  | 1.502 (1.190-1.896) | 0.001* |  | 1.448 (1.135-1.845) | 0.003* |
| Q1 | 1 (Reference) |  |  | 1 (Reference) |  |  | 1 (Reference) |  |
| Q2 | 1.661 (1.182-2.333) | 0.003* |  | 1.549 (1.095-2.189) | 0.013* |  | 1.496 (1.054-2.125) | 0.024* |
| Q3 | 1.829 (1.286-2.600) | 0.001* |  | 1.651 (1.146-2.377) | 0.007* |  | 1.554 (1.070-2.257) | 0.021* |
|  |  |  |  |  |  |  |  |  |
| Baseline TyG-BMI | 1.009 (1.005-1.012) | <0.001* |  | 1.008 (1.004-1.012) | <0.001* |  | 1.008 (1.004-1.012) | <0.001* |
| Q1 | 1 (Reference) |  |  | 1 (Reference) |  |  | 1 (Reference) |  |
| Q2 | 1.565 (1.092-2.244) | 0.015* |  | 1.559 (1.085-2.241) | 0.016* |  | 1.526 (1.060-2.197) | 0.023* |
| Q3 | 2.342 (1.652-3.319) | <0.001* |  | 2.317 (1.626-3.301) | <0.001* |  | 2.256 (1.574-3.232) | <0.001* |
|  |  |  |  |  |  |  |  |  |
| Mean TyG index | 2.150 (1.652-2.797) | <0.001* |  | 2.108 (1.529-2.663) | <0.001* |  | 1.974 (1.485-2.625) | <0.001* |
| Q1 | 1 (Reference) |  |  | 1 (Reference) |  |  | 1 (Reference) |  |
| Q2 | 1.860 (1.300-2.661) | 0.001* |  | 1.778 (1.236-2.556) | 0.002* |  | 1.730 (1.200-2.494) | 0.003* |
| Q3 | 2.645 (1.848-3.786) | <0.001* |  | 2.416 (1.662-3.511) | <0.001* |  | 2.335 (1.599-3.409) | <0.001* |
|  |  |  |  |  |  |  |  |  |
| TyG-SD | 2.743 (1.923-3.912) | <0.001* |  | 2.563 (1.782-3.686) | <0.001* |  | 2.768 (1.884-4.067) | <0.001* |
| Q1 | 1 (Reference) |  |  | 1 (Reference) |  |  | 1 (Reference) |  |
| Q2 | 1.297 (0.911-1.847) | 0.149 |  | 1.271 (0.892-1.811) | 0.184 |  | 1.256 (0.880-1.791) | 0.209 |
| Q3 | 2.010 (1.435-2.815) | <0.001* |  | 1.966 (1.403-2.756) | <0.001* |  | 1.933 (1.376-2.713) | <0.001* |
|  |  |  |  |  |  |  |  |  |
| **Diabetes** |  |  |  |  |  |  |  |  |
| Baseline TyG index | 1.611 (1.304-1.992) | <0.001* |  | 1.551 (1.244-1.934) | <0.001* |  | 1.535 (1.223-1.927) | <0.001* |
| Q1 | 1 (Reference) |  |  | 1 (Reference) |  |  | 1 (Reference) |  |
| Q2 | 1.487 (0.855-2.585) | 0.160 |  | 1.333 (0.763-2.329) | 0.312 |  | 1.234 (0.703-2.168) | 0.464 |
| Q3 | 2.152 (1.308-3.540) | <0.001* |  | 1.931 (1.163-3.205) | 0.011* |  | 1.765 (1.054-2.953) | 0.031* |
|  |  |  |  |  |  |  |  |  |
| Baseline TyG-BMI | 1.011 (1.007-1.015) | <0.001* |  | 1.011 (1.007-1.015) | <0.001* |  | 1.011 (1.007-1.015) | <0.001* |
| Q1 | 1 (Reference) |  |  | 1 (Reference) |  |  | 1 (Reference) |  |
| Q2 | 1.791 (1.001-3.202) | 0.049* |  | 1.837 (1.026-3.288) | 0.041* |  | 1.904 (1.063-3.410) | 0.030* |
| Q3 | 3.191 (1.872-5.439) | <0.001* |  | 3.206 (1.878-5.472) | <0.001* |  | 3.078 (1.799-5.265) | <0.001* |
|  |  |  |  |  |  |  |  |  |
| Mean TyG index | 2.399 (1.835-3.136) | <0.001* |  | 2.180 (1.644-2.892) | <0.001* |  | 2.257 (1.687-3.021) | <0.001* |
| Q1 | 1 (Reference) |  |  | 1 (Reference) |  |  | 1 (Reference) |  |
| Q2 | 2.305 (1.176-4.520) | 0.015* |  | 2.209 (1.124-4.340) | 0.021* |  | 2.029 (1.027-4.008) | 0.042* |
| Q3 | 3.506 (1.877-6.549) | <0.001* |  | 3.112 (1.654-5.855) | <0.001* |  | 2.954 (1.563-5.581) | 0.001* |
|  |  |  |  |  |  |  |  |  |
| TyG-SD | 2.246 (1.205-4.186) | 0.011* |  | 2.193 (1.161-4.144) | 0.016* |  | 2.558 (1.326-4.935) | 0.005* |
| Q1 | 1 (Reference) |  |  | 1 (Reference) |  |  | 1 (Reference) |  |
| Q2 | 1.742 (0.995-3.049) | 0.052 |  | 1.761 (1.005-3.084) | 0.048* |  | 1.866 (1.050-3.314) | 0.033* |
| Q3 | 1.953 (1.158-3.294) | 0.012* |  | 1.977 (1.171-3.339) | 0.011* |  | 2.157 (1.259-3.695) | 0.005* |

**Model 1**: Adjusted for age and gender (male or female).

**Model 2**: Adjusted for age, gender (male or female), baseline BMI, smoke (never, former, and current), drink (never, former, and current), hypertension (yes or no), and diabetes (yes or no).

**Model 3**: Adjusted for age, gender (male or female), baseline BMI, smoke (never, former, and current), drink (never, former, and current), hypertension (yes or no), and diabetes (yes or no), prior AMI (yes or no), baseline LDL-C, multivessel lesion (yes or no), LVEF, and medications (administration of ACEI/ARB, antidiabetic agents, antihypertensive agents, and β-receptor blocker) (yes or no).

The four TyG indicators were analyzed as continuous variables in Cox regression models. Moreover, the study also stratified participants into tertiles based on four TyG indicators (baseline TyG index, baseline TyG-BMI, mean TyG index, and TyG-SD), with the designation Q1<Q2<Q3, using Q1 as the reference group. Given the significant collinearity observed between the TyG-BMI and the BMI, the model employed at baseline TyG-BMI excludes the BMI as a variable. TyG: triglyceride-glucose; TyG-BMI: triglyceride glucose-body mass index; TyG-SD: triglyceride glucose index-standard deviation; HR: hazard ratios; CI: confidence interval; BMI: body mass index; AMI: acute myocardial infarction; LDL-C: low density lipoprotein cholesterol; LVEF: left ventricular ejection fraction; ACEI: angiotensin converting enzyme inhibitor; ARB: angiotensin receptor blocker; *: P < 0.05.

**Supplementary Table 3** Subgroup analysis according to stratification of hypertension

| Variables | Model 1 |  |  | Model 2 |  |  | Model 3 |  |
| --- | --- | --- | --- | --- | --- | --- | --- | --- |
|  | Adjusted HR (95%CI) | *P* value |  | Adjusted HR (95%CI) | *P* value |  | Adjusted HR (95%CI) | *P* value |
| **No hypertension** |  |  |  |  |  |  |  |  |
| Baseline TyG index | 1.883 (1.471-2.410) | <0.001* |  | 1.700 (1.294-2.234) | <0.001* |  | 1.587 (1.199-2.100) | 0.001* |
| Q1 | 1 (Reference) |  |  | 1 (Reference) |  |  | 1 (Reference) |  |
| Q2 | 2.082 (1.216-3.562) | 0.007* |  | 1.894 (1.097-3.270) | 0.022* |  | 1.976 (1.127-3.465) | 0.017* |
| Q3 | 2.639 (1.566-4.447) | <0.001* |  | 2.151 (1.246-3.715) | 0.006* |  | 2.139 (1.208-3.787) | 0.009* |
|  |  |  |  |  |  |  |  |  |
| Baseline TyG-BMI | 1.012 (1.007-1.017) | <0.001* |  | 1.011 (1.005-1.016) | <0.001* |  | 1.010 (1.004-1.016) | 0.001* |
| Q1 | 1 (Reference) |  |  | 1 (Reference) |  |  | 1 (Reference) |  |
| Q2 | 2.081 (1.208-3.583) | 0.008* |  | 1.889 (1.092-3.269) | 0.023* |  | 1.884 (1.086-3.268) | 0.024* |
| Q3 | 3.384 (1.990-5.757) | <0.001* |  | 2.998 (1.740-5.165) | <0.001* |  | 3.039 (1.740-5.309) | <0.001* |
|  |  |  |  |  |  |  |  |  |
| Mean TyG index | 2.441 (1.754-3.396) | <0.001* |  | 2.136 (1.479-3.085) | <0.001* |  | 2.073 (1.426-3.012) | <0.001* |
| Q1 | 1 (Reference) |  |  | 1 (Reference) |  |  | 1 (Reference) |  |
| Q2 | 1.920 (1.124-3.280) | 0.017* |  | 1.680 (0.973-2.902) | 0.063 |  | 1.676 (0.963-2.918) | 0.068 |
| Q3 | 2.792 (1.656-4.707) | <0.001* |  | 2.202 (1.257-3.858) | 0.006* |  | 2.289 (1.290-4.060) | 0.005* |
|  |  |  |  |  |  |  |  |  |
| TyG-SD | 7.853 (3.620-17.039) | <0.001* |  | 5.760 (2.489-13.332) | <0.001* |  | 5.008 (2.041-12.287) | <0.001* |
| Q1 | 1 (Reference) |  |  | 1 (Reference) |  |  | 1 (Reference) |  |
| Q2 | 1.597 (0.899-2.837) | 0.110 |  | 1.520 (0.853-2.711) | 0.156 |  | 1.497 (0.836-2.682) | 0.175 |
| Q3 | 2.550 (1.504-4.322) | <0.001* |  | 2.185 (1.269-3.762) | 0.005* |  | 1.963 (1.127-3.422) | 0.017* |
|  |  |  |  |  |  |  |  |  |
| **Hypertension** |  |  |  |  |  |  |  |  |
| Baseline TyG index | 1.750 (1.457-2.101) | <0.001* |  | 1.473 (1.212-1.790) | <0.001* |  | 1.447 (1.182-1.771) | <0.001* |
| Q1 | 1 (Reference) |  |  | 1 (Reference) |  |  | 1 (Reference) |  |
| Q2 | 1.485 (1.055-2.089) | 0.023* |  | 1.321 (0.934-1.868) | 0.115 |  | 1.274 (0.898-1.808) | 0.175 |
| Q3 | 2.088 (1.514-2.881) | <0.001* |  | 1.641 (1.171-2.299) | 0.004* |  | 1.508 (1.069-2.128) | 0.019* |
|  |  |  |  |  |  |  |  |  |
| Baseline TyG-BMI | 1.011 (1.007-1.014) | <0.001* |  | 1.009 (1.006-1.013) | <0.001* |  | 1.009 (1.006-1.013) | <0.001* |
| Q1 | 1 (Reference) |  |  | 1 (Reference) |  |  | 1 (Reference) |  |
| Q2 | 1.509 (1.049-2.172) | 0.027* |  | 1.452 (1.008-2.091) | 0.045* |  | 1.451 (1.006-2.092) | 0.046* |
| Q3 | 2.609 (1.869-3.640) | <0.001* |  | 2.389 (1.706-3.344) | <0.001* |  | 2.346 (1.670-3.294) | <0.001* |
|  |  |  |  |  |  |  |  |  |
| Mean TyG index | 2.571 (2.080-3.177) | <0.001* |  | 2.146 (1.698-2.712) | <0.001* |  | 2.163 (1.698-2.756) | <0.001* |
| Q1 | 1 (Reference) |  |  | 1 (Reference) |  |  | 1 (Reference) |  |
| Q2 | 2.023 (1.375-2.976) | <0.001* |  | 1.876 (1.272-2.766) | 0.001* |  | 1.824 (1.234-2.698) | 0.003* |
| Q3 | 3.415 (2.378-4.904) | <0.001* |  | 2.699 (1.852-3.933) | <0.001* |  | 2.582 (1.767-3.774) | <0.001* |
|  |  |  |  |  |  |  |  |  |
| TyG-SD | 2.542 (1.833-3.525) | <0.001* |  | 2.087 (1.431-3.044) | <0.001* |  | 2.250 (1.528-3.314) | <0.001* |
| Q1 | 1 (Reference) |  |  | 1 (Reference) |  |  | 1 (Reference) |  |
| Q2 | 1.476 (1.049-2.078) | 0.025* |  | 1.388 (0.985-1.955) | 0.061 |  | 1.392 (0.986-1.966) | 0.060 |
| Q3 | 2.149 (1.561-2.958) | <0.001* |  | 1.800 (1.298-2.497) | <0.001* |  | 1.834 (1.319-2.550) | <0.001* |

**Model 1**: Adjusted for age and gender (male or female).

**Model 2**: Adjusted for age, gender (male or female), baseline BMI, smoke (never, former, and current), drink (never, former, and current), hypertension (yes or no), and diabetes (yes or no).

**Model 3**: Adjusted for age, gender (male or female), baseline BMI, smoke (never, former, and current), drink (never, former, and current), hypertension (yes or no), and diabetes (yes or no), prior AMI (yes or no), baseline LDL-C, multivessel lesion (yes or no), LVEF, and medications (administration of ACEI/ARB, antidiabetic agents, antihypertensive agents, and β-receptor blocker) (yes or no).

The four TyG indicators were analyzed as continuous variables in Cox regression models. Moreover, the study also stratified participants into tertiles based on four TyG indicators (baseline TyG index, baseline TyG-BMI, mean TyG index, and TyG-SD), with the designation Q1<Q2<Q3, using Q1 as the reference group. Given the significant collinearity observed between the TyG-BMI and the BMI, the model employed at baseline TyG-BMI excludes the BMI as a variable. TyG: triglyceride-glucose; TyG-BMI: triglyceride glucose-body mass index; TyG-SD: triglyceride glucose index-standard deviation; HR: hazard ratios; CI: confidence interval; BMI: body mass index; AMI: acute myocardial infarction; LDL-C: low density lipoprotein cholesterol; LVEF: left ventricular ejection fraction; ACEI: angiotensin converting enzyme inhibitor; ARB: angiotensin receptor blocker; *: P < 0.05.

**Supplementary Table 4** Subgroup analysis according to stratification of LDL-C levels

| Variables | Model 1 |  |  | Model 2 |  |  | Model 3 |  |
| --- | --- | --- | --- | --- | --- | --- | --- | --- |
|  | Adjusted HR (95%CI) | *P* value |  | Adjusted HR (95%CI) | *P* value |  | Adjusted HR (95%CI) | *P* value |
| **LDL-C <1.8** |  |  |  |  |  |  |  |  |
| Baseline TyG index | 1.777 (1.394-2.265) | <0.001* |  | 1.532 (1.179-1.992) | 0.001* |  | 1.473 (1.124-1.929) | 0.005* |
| Q1 | 1 (Reference) |  |  | 1 (Reference) |  |  | 1 (Reference) |  |
| Q2 | 1.799 (1.149-2.816) | 0.010* |  | 1.538 (0.971-2.437) | 0.067 |  | 1.559 (0.982-2.476) | 0.060 |
| Q3 | 2.431 (1.569-3.767) | <0.001* |  | 1.883 (1.185-2.992) | 0.007* |  | 1.783 (1.116-2.848) | 0.016* |
|  |  |  |  |  |  |  |  |  |
| Baseline TyG-BMI | 1.010 (1.005-1.014) | <0.001* |  | 1.008 (1.003-1.013) | 0.001* |  | 1.008 (1.003-1.013) | 0.002* |
| Q1 | 1 (Reference) |  |  | 1 (Reference) |  |  | 1 (Reference) |  |
| Q2 | 1.496 (0.941-2.379) | 0.089 |  | 1.321 (0.825-2.114) | 0.246 |  | 1.287 (0.802-2.064) | 0.296 |
| Q3 | 2.203 (1.418-3.420) | <0.001* |  | 1.903 (1.220-2.971) | 0.005* |  | 1.814 (1.153-2.853) | 0.010* |
|  |  |  |  |  |  |  |  |  |
| Mean TyG index | 2.581 (1.919-3.472) | <0.001* |  | 2.208 (1.581-3.085) | <0.001* |  | 2.122 (1.498-3.007) | <0.001* |
| Q1 | 1 (Reference) |  |  | 1 (Reference) |  |  | 1 (Reference) |  |
| Q2 | 1.902 (1.158-3.126) | 0.011* |  | 1.735 (1.050-2.867) | 0.031* |  | 1.676 (1.012-2.778) | 0.045* |
| Q3 | 3.209 (2.023-5.090) | <0.001* |  | 2.489 (1.525-4.064) | <0.001* |  | 2.329 (1.416-3.831) | 0.001* |
|  |  |  |  |  |  |  |  |  |
| TyG-SD | 2.683 (1.885-3.819) | <0.001* |  | 2.404 (1.592-3.632) | <0.001* |  | 2.498 (1.601-3.898) | <0.001* |
| Q1 | 1 (Reference) |  |  | 1 (Reference) |  |  | 1 (Reference) |  |
| Q2 | 0.930 (0.558-1.552) | 0.781 |  | 0.838 (0.501-1.402) | 0.501 |  | 0.786 (0.467-1.324) | 0.366 |
| Q3 | 2.414 (1.578-3.693) | <0.001* |  | 1.914 (1.231-2.975) | 0.004* |  | 1.837 (1.168-2.888) | 0.008* |
|  |  |  |  |  |  |  |  |  |
| **LDL-C ≥1.8** |  |  |  |  |  |  |  |  |
| Baseline TyG index | 1.802 (1.504-2.158) | <0.001* |  | 1.483 (1.211-1.816) | <0.001* |  | 1.486 (1.210-1.824) | <0.001* |
| Q1 | 1 (Reference) |  |  | 1 (Reference) |  |  | 1 (Reference) |  |
| Q2 | 1.589 (1.090-2.317) | 0.016* |  | 1.358 (0.927-1.989) | 0.117 |  | 1.334 (0.907-1.961) | 0.143 |
| Q3 | 2.186 (1.535-3..113) | <0.001* |  | 1.596 (1.100-2.315) | 0.014* |  | 1.553 (1.066-2.263) | 0.022* |
|  |  |  |  |  |  |  |  |  |
| Baseline TyG-BMI | 1.012 (1.009-1.016) | <0.001* |  | 1.011 (1.007-1.014) | <0.001* |  | 1.010 (1.007-1.014) | <0.001* |
| Q1 | 1 (Reference) |  |  | 1 (Reference) |  |  | 1 (Reference) |  |
| Q2 | 1.855 (1.243-2.768) | 0.002* |  | 1.752 (1.171-2.620) | 0.006* |  | 1.748 (1.169-2.613) | 0.007* |
| Q3 | 3.467 (2.397-5.013) | <0.001* |  | 3.006 (2.056-4.396) | <0.001* |  | 2.980 (2.036-4.362) | <0.001* |
|  |  |  |  |  |  |  |  |  |
| Mean TyG index | 2.558 (2.054-3.185) | <0.001* |  | 2.088 (1.635-2.667) | <0.001* |  | 2.143 (1.667-2.754) | <0.001* |
| Q1 | 1 (Reference) |  |  | 1 (Reference) |  |  | 1 (Reference) |  |
| Q2 | 2.042 (1.365-3.053) | 0.001* |  | 1.768 (1.174-2.660) | 0.006* |  | 1.745 (1.157-2.631) | 0.008* |
| Q3 | 3.370 (2.301-4.937) | <0.001* |  | 2.513 (1.679-3.763) | <0.001* |  | 2.491 (1.660-3.737) | <0.001* |
|  |  |  |  |  |  |  |  |  |
| TyG-SD | 4.141 (2.363-7.256) | <0.001* |  | 2.759 (1.521-5.005) | 0.001* |  | 2.855 (1.542-5.288) | 0.001* |
| Q1 | 1 (Reference) |  |  | 1 (Reference) |  |  | 1 (Reference) |  |
| Q2 | 1.917 (1.329-2.766) | <0.001* |  | 1.817 (1.257-2.625) | 0.001* |  | 1.861 (1.285-2.696) | 0.001* |
| Q3 | 2.182 (1.527-3.119) | <0.001* |  | 1.909 (1.324-2.754) | 0.001* |  | 1.901 (1.312-2.754) | 0.001* |

**Model 1**: Adjusted for age and gender (male or female).

**Model 2**: Adjusted for age, gender (male or female), baseline BMI, smoke (never, former, and current), drink (never, former, and current), hypertension (yes or no), and diabetes (yes or no).

**Model 3**: Adjusted for age, gender (male or female), baseline BMI, smoke (never, former, and current), drink (never, former, and current), hypertension (yes or no), and diabetes (yes or no), prior AMI (yes or no), baseline LDL-C, multivessel lesion (yes or no), LVEF, and medications (administration of ACEI/ARB, antidiabetic agents, antihypertensive agents, and β-receptor blocker) (yes or no).

The four TyG indicators were analyzed as continuous variables in Cox regression models. Moreover, the study also stratified participants into tertiles based on four TyG indicators (baseline TyG index, baseline TyG-BMI, mean TyG index, and TyG-SD), with the designation Q1<Q2<Q3, using Q1 as the reference group. Given the significant collinearity observed between the TyG-BMI and the BMI, the model employed at baseline TyG-BMI excludes the BMI as a variable. TyG: triglyceride-glucose; TyG-BMI: triglyceride glucose-body mass index; TyG-SD: triglyceride glucose index-standard deviation; HR: hazard ratios; CI: confidence interval; BMI: body mass index; AMI: acute myocardial infarction; LDL-C: low density lipoprotein cholesterol; LVEF: left ventricular ejection fraction; ACEI: angiotensin converting enzyme inhibitor; ARB: angiotensin receptor blocker; *: P < 0.05.
